# Astrocyte reactivity across the AD continuum measured by [^18^F]SMBT-1 and its relationship with the Aβ burden

**DOI:** 10.1007/s00259-026-07824-3

**Published:** 2026-05-06

**Authors:** Yingying Wu, Kotaro Hiraoka, Berihu Mesfin, Asuka Kikuchi, Shoichi Watanuki, Shunji Mugikura, Naoki Tomita, Aiko Ishiki, Katsutoshi Furukawa, Yoshihito Funaki, Jun Toyohara, Yasuyuki Kimura, Ryuichi Harada, Shozo Furumoto, Akio Kikuchi, Hiroshi Watabe, Ryota Kobayashi, Takashi Nihashi, Takashi Kato, Kenji Ishii, Shinobu Kawakatsu, Nobuyuki Okamura, Manabu Tashiro, SMBTSD study project group*, Yoichi Ishikawa, Kazuko Takeda, Kazuhiko Yanai, Miho Shidahara, Masayasu Miyake, Mizue Kusaba, Kei Takase, Taizen Nakase, Tatsushi Mutoh, Yasuyuki Taki, Mari Otshuki, Tadaho Nakamura, Fumito Naganuma, Tomonori Matsuura, Kenji Ishibashi, Tetsuro Tago, Muneyuki Sakata, Yuto Kamitaka, Etsuko Imabayashi, Masashi Kameyama, Mika Tanaka, Keita Sakurai, Kengo Ito, Akinori Nakamura, Keisuke Suzuki, Masashi Tsujimoto, Hiroshi Ikenuma, Junichiro Abe, Kaori Iwata, Daichi Morioka, Masafumi Kanoto, Kazukuni Kirii, Kenichi Utano

**Affiliations:** 1 Research Center for Accelerator and Radioisotope Science (RARiS), Tohoku University, 6-3 Aramaki Aza Aoba, Aoba-ku, Sendai, Miyagi 980-8578 Japan; 2 Tohoku University Hospital, Tohoku University, Sendai, Japan; 3 Tohoku Medical and Pharmaceutical University, Sendai, Japan; 4 Tokyo Metropolitan Institute for Geriatrics and Gerontology, Tokyo, Japan; 5 National Center for Geriatrics and Gerontology, Obu, Japan; 6 Yamagata University Hospital, Yamagata, Japan; 7 Aizu Medical Center, Fukushima Medical University, Aizuwakamatsu, Japan; 8 Yamagata Prefectural University of Health Sciences, Yamagata, Japan

**Keywords:** Alzheimer's disease, Mild cognitive impairment (MCI), Monoamine oxidase B (MAO-B), Reactive astrogliosis, Amyloid-β (Aβ), [^18^F]SMBT-1

## Abstract

**Purpose:**

Astrocytes colocalize with fibrillar amyloid-β (Aβ) plaques in postmortem Alzheimer’s disease (AD) brain tissue; however, their spatiotemporal dynamics in vivo remain poorly understood. This multicenter study aimed to investigate the progression of astrocyte reactivity across the AD continuum, including healthy controls (HC), mild cognitive impairment (MCI), and AD, using the novel monoamine oxidase B (MAO-B)-specific PET tracer [^18^F]SMBT-1, while exploring its association with cognitive performance and amyloid burden.

**Methods:**

A total of 91 participants (35 HC, 44 MCI, 12 AD) underwent [^18^F]SMBT-1 PET, amyloid PET, T1-weighted MRI, and standardized neuropsychological assessments. Standardized uptake value ratios (SUVRs) were calculated based on [^18^F]SMBT-1 PET data using four reference regions for subgroup comparisons stratified by Aβ status.

**Results:**

[^18^F]SMBT-1 uptake was significantly elevated in amyloid-positive MCI (MCI+) and AD groups compared with amyloid-negative HC (HC−) in the frontal, temporal, and posterior cingulate regions. Notably, astrogliosis patterns distinguished MCI subtypes: MCI+ individuals exhibited a widespread AD-like pattern, whereas the MCI− group showed a distinct profile. Furthermore, the uptake in symptomatic MCI+ individuals was significantly higher than that in asymptomatic HC+ individuals. Regional SMBT-1 uptake also strongly correlated with greater Aβ burden and worse cognitive scores.

**Conclusion:**

This study demonstrates that [^18^F]SMBT-1 is a promising tool for characterizing the spatial pattern and magnitude of reactive astrogliosis across the Aβ-defined AD continuum. Our findings further suggest that astrogliosis may represent an important mechanistic link between amyloid pathology and cognitive impairment, supporting its potential relevance in therapeutic development.

**Clinical trial registration:**

Japan Registry of Clinical Trials (jRCT) jRCTs031210602, registered Feb. 07, 2022.

**URL for the trial registry:**

https://jrct.mhlw.go.jp/en-latest-detail/jRCTs031210602.

**Supplementary Information:**

The online version contains supplementary material available at 10.1007/s00259-026-07824-3.

## Introduction

Alzheimer’s disease (AD) is a neurodegenerative disorder characterized by progressive cognitive decline, with hallmark pathologies including amyloid-beta (Aβ) plaques, tau tangles, reactive astrocytes, microgliosis, and neuronal loss [1]. Astrocytes are the most abundant glial cell population in the brain and play an important role in maintaining synaptic homeostasis by regulating synaptic function, calcium signaling, and brain metabolism [2]. This form of glial cell has been proposed to play an important role early in the pathophysiology of AD and exacerbates disease effects when they are reactive because of neuroinflammation [3, 4]. And astrocyte reactivity can increase the expression of inflammatory mediators, reactive oxygen species, and Aβ deposition in mouse models [5]. Postmortem studies of AD brains have demonstrated abundant reactive astrocytes and microglia around Aβ plaques [6, 7], but the extent to which this is a consequence or contributing factor for the formation of Aβ remains uncertain.

Monoamine oxidase B (MAO-B) is primarily expressed in astrocytes. Furthermore, MAO-B levels have been shown to increase in the brains of patients with sporadic and familial AD and mild cognitive impairment (MCI) [8, 9]. A recent study showed that MAO-B is elevated in AD pyramidal neurons, associated with γ-secretase, and regulates neuronal Aβ-peptide levels [10]. Reactive astrocytes and MAO-B have emerged as potential targets for the diagnosis and treatment of AD [11].

Molecular neuroimaging studies have developed several positron emission tomography (PET) tracers to study reactive astrocytes. Traditional tracers, such as the irreversible MAO-B tracers [^11^C]deuterium-L-deprenyl (DED) and [^18^F]F-DED [8, 12, 13], substrate-based MAO-B tracer [^11^C]Cou [14], and mitochondrial imidazoline 2 binding site (I2BS) tracer [^11^C]BU99008, have been employed as surrogate markers of astrogliosis [15]. Tracers such as [^11^C]DED have demonstrated that reactive astrogliosis can occur in the prodromal stages of both sporadic and familial AD [8, 16]. In animal models (e.g., APPswe and PS2APP), reactive astrocytes measured with [^11^C]DED and [^18^F]F-DED were shown to precede the increase in amyloid-PET signals [9, 12, 13]. However, [^11^C]DED and [^11^C]BU99008 are labeled with carbon-11, which has a short half-life (approximately 20 min) that limits their widespread clinical and research applications. The need for tracers with longer half-lives and higher specificity remains unmet.

Recently, a novel fluorine-18-labeled MAO-B tracer, [^18^F]SMBT-1, was developed to address these challenges. [^18^F]SMBT-1 exhibited a high in vitro binding affinity (KD = 3.5 nM) and selectivity for MAO-B​​. It demonstrates robust brain entry and reversible binding kinetics, with more than 85% of its signal blocked by the selective MAO-B inhibitor selegiline [17], indicating low non-specific binding​​. The regional binding of [^18^F]SMBT-1 closely aligned with the known brain distribution of MAO-B (R^2^ = 0.84) [17]. 

Cross-sectional studies have demonstrated significant regional binding increases in areas such as the posterior cingulate, supramarginal gyrus, and lateral occipital cortex, validating [^18^F]SMBT-1 as a promising surrogate marker for reactive astrogliosis in AD, for which SMBT-1 PET findings in the AD spectrum have been reported [18]. However, that study included a relatively small number of MCI participants and did not report detailed pair-wise comparisons between MCI with (MCI+) and without (MCI−) amyloid β burden and AD subgroups. Characterizing the astrogliosis profile within these biologically defined groups is essential for understanding the astrocytic mechanisms underlying early AD pathophysiology. In the present study, we addressed this gap by including a larger and well-characterized cohort, particularly within the MCI population, enabling us to analyze reactive astrogliosis within five biologically defined groups based on amyloid status: healthy controls (cognitively normal) without Aβ burden (HC−), healthy controls with Aβ burden (HC+), MCI−, MCI+, and AD. This allowed voxel-wise and region-based analyses to characterize astrocyte reactivity patterns across a biologically defined AD continuum, according to the 2024 NIA-AA Revised Criteria for Diagnosis and Staging of Alzheimer’s Disease [19], and contrasts these patterns with key amyloid-negative comparison groups (HC − and MCI−). Furthermore, we examined the relationship between regional [^18^F]SMBT-1 binding and amyloid burden as well as cognitive performance.

A second critical issue in [^18^F]SMBT-1 PET imaging is the optimal strategy for semiquantitative calculations. Various “pseudo-reference” regions have been proposed based on kinetic modeling and first-in-human studies, including the subcortical white matter (SWM) and three cerebellar references: cerebellar grey matter (CGM), cerebellar white matter (CWM), and the whole cerebellum (WC) [17, 20, 21]. However, these studies also suggested that all candidate regions may exhibit some degree of specific binding, and different studies have recommended different optimal candidates. Therefore, no consensus has been reached regarding the best reference region for clinical evaluation. Therefore, the secondary aim of the current study was to empirically examine the usefulness of these different references in distinguishing the clinical subgroups of the AD continuum in our cohort.

## Participants and Methods

This study was a multicenter research project (Study on the usefulness of [^18^F]SMBT-1 in the stratification of dementia: SMBTSD study) conducted at several institutions across Japan, including Tohoku University Hospital (and its Aobayama branch for clinical research), Tohoku Medical and Pharmaceutical University Hospital, Aizu Medical Center of Fukushima Medical University, Yamagata University Hospital, Tokyo Metropolitan Institute for Geriatrics and Gerontology, and the National Center for Geriatrics and Gerontology. To ensure geographical diversity, participant data were collected from six hospitals and institutes belonging to the following three districts: the Tohoku region (northeast region), Kanto region (Tokyo metropolitan area), and Chukyo region (Aichi prefectural area). [^18^F]SMBT-1 PET was performed at the Aobayama branch of Tohoku University Hospital, Tokyo Metropolitan Institute for Geriatrics and Gerontology, and National Center for Geriatrics and Gerontology.

### Subjects

This study included 91 participants from three groups: 35 HC subjects, 44 MCI subjects, and 12 AD subjects. All participants, aged between 50 and 90 years, provided written informed consent. Participants using irreversible MAO-B inhibitors (e.g., Selegiline, Rasagiline) and habitual smokers were excluded from this study. MCI was diagnosed according to the criteria established by Petersen et al. [22]. AD was diagnosed as probable AD dementia with a high biomarker probability according to NIA-AA 2011 [23]. The Tokyo Metropolitan Geriatric Medical Center Clinical Research Review Board approved this study protocol, and the inclusion and exclusion criteria are provided in Online Resource 1.

### Neuropsychological assessments

All participants underwent neuropsychological assessments to evaluate cognitive functions, taking a total time of approximately 70 min for the Mini-Mental State Examination (MMSE, for 10 min), the Japanese version of the Alzheimer’s Disease Assessment Scale-Cognitive Subscale (ADAS-Jcog, for 30 min), and Wechsler Memory Scale Logical Memory II (WMS-LMII, for 30 min). However, only 74 participants completed the ADAS-Jcog assessment.

### Image acquisition

All PET scans were acquired using PET/CT scanners, including Discovery MI and Discovery 710 (GE Healthcare, Chicago, USA), Biograph True Point and Biograph mCT (Siemens Healthineers, Erlangen, Germany), and a dedicated PET scanner, Eminence-B (SET-3000B/X: Shimadzu Corporation, Kyoto, Japan). To ensure data harmonization and comparability across these different scanners, standardized protocols were implemented, which were established by the Japanese trial-ready cohort study (J-TRC consortium: https://www.j-trc.org/ja/welcome). For attenuation correction, low-dose CT was performed on the PET/CT scanners, while the dedicated PET scanner employed a transmission source.

### SMBT-1 PET

[^18^F]SMBT-1 was manufactured at the Research Center for Accelerator and Radioisotope Science of Tohoku University (adjunct to the Tohoku University Hospital Aobayama branch), Tokyo Metropolitan Institute for Geriatrics and Gerontology, and National Center for Geriatrics and Gerontology [24, 25]. The manufacturing process adhered to internal Quality Control Standards (Quality Control Standards established by the Tohoku University Research Center for Accelerator and Radioisotope Science) to ensure consistent quality under a controlled production environment. For astroglial PET imaging, a single intravenous injection of [^18^F]SMBT-1 (110–260 MBq, approximately 3.0–7.0 mCi) was administered, followed by PET imaging for 30 min (6 × 300 s) between 60 and 90 min post-injection.

### Aβ PET

For Aβ PET imaging, either [^11^C]PiB or [^18^F]flutemetamol was used as Aβ PET tracer. A single intravenous injection of [^11^C]PiB (185–740 MBq, 5–20 mCi) was administered, followed by PET imaging for 20 min (4 × 300 s) between 50 and 70 min post-injection. Alternatively, a single intravenous injection of [^18^F]flutemetamol (110–260 MBq, 3.0–7.0 mCi) was administered, followed by PET imaging for 20–30 min (4–6 × 300 s) between 90 and 120 min post-injection.

To harmonize quantitative data from these two tracers and ensure comparability, all individual uptake values were converted to the standardized centiloid scale using PMOD [26–28].

### MRI

High-resolution 3D brain morphology imaging using 3D T1-weighted sequences (scan duration: approximately 10 min) was performed for all the subjects. These images were used for both the initial screening and subsequent PET data analysis.

### Image analysis

After the PET data were preprocessed using PMOD version 4.3 (Bruker Preclinical Imaging, Fallanden, Switzerland), a uniform image-processing procedure was applied to ensure data consistency. These included reorientation, centering, frame averaging, and standardized uptake value (SUV) calculations. The processed PET data were then used for subsequent analyses.

First, using SPM12 (Wellcome Trust Center for Neuroimaging, London, UK), PET images of each participant were co-registered with their corresponding T1-weighted MR images. This step ensured precise alignment of the structural and functional data, creating a PET image in the native MRI space for each participant. Aβ PET images of these participants were processed in a similar manner.

Subsequently, both the co-registered PET and MRI images were normalized to the Montreal Neurological Institute (MNI) space using the PMOD software. These maps were applied directly to PMOD without additional thresholding because the built-in segmentation algorithm provided robust results. For region of interest (ROI) analysis, using the standard AAL ROI template, the volumes of interest (VOIs) were defined in normalized space. The inverse transformation parameters from the normalization process were then used to warp the VOIs back to the native PET space, enabling precise sampling of regional SUV values for approximately 95 grey matter regions (not including those for cerebellum). In addition to the cortical regions, exploratory white matter (WM) ROI analysis was also performed. The frontal, parietal, occipital, temporal, and insular WM regions were defined using the AAL atlas implemented in PMOD. SUVs were extracted from each WM ROI and normalized to CWM to calculate SUVRs in a similar manner to that for grey matter regions.

To streamline the analysis, a pipeline was developed in PMOD, which consistently applied these settings across all participants. This automated process ensured the efficient extraction of regional SUV data.

Finally, the SUV data were normalized to the following reference regions: SWM and three cerebellar references: CWM, CGM, and WC. This normalization yielded standardized uptake value ratio (SUVR) values for each VOI, which were used for statistical analysis and group comparisons. Previous studies [17, 20, 21] have recommended both SWM and CGM as pseudo-reference regions for MAO-B PET quantification. Given these mixed recommendations, we compared SUVR differences across the reference regions in our preliminary analysis to observe inter-subgroup differences. Amyloid positivity was defined by visual assessment, while centiloid values were used for supportive analysis (< 10 for negative, > 30 for positive) [29, 30].

### Statistical analysis

#### Demographic analysis

The demographic and clinical characteristics were compared across the five diagnostic groups. Continuous variables (e.g., age, education, and cognitive scores) were analyzed using one-way Analysis of Variance (ANOVA), followed by Bonferroni correction for post-hoc tests for pairwise comparisons. Categorical variables (e.g., sex) were analyzed using the chi-square (χ²) test. Statistical significance was set at *p* < 0.05.

#### ROI data analysis

Repeated analysis of variance (ANOVA) with Bonferroni correction for post-hoc analysis was used to compare subgroups such as HC−, HC+, MCI−, MCI+, and AD in each brain region (IBM SPSS Statistics 27). Statistical significance was set at *p* < 0.05. The data are presented as mean± standard deviation (S.D.). Similar statistical analyses were performed both on grey matter and WM regions.

Selection of an appropriate reference region is critical for accurate SUVR quantification in PET studies. A first-in-human PET study suggested the use of SWM, the whole cerebellum (WC), and CWM as potential reference regions for MAO-B PET imaging [17]. Later, kinetic studies demonstrated that CGM can be used as a reference and that CGM can be one of the best candidate reference regions [20, 21]. Therefore, we examined the SUVs of cerebellar references, such as CWM, CGM, and WC, as well as those of SWM. Group differences were observed in each of the four reference regions. Correlations were analyzed using the Pearson’s correlation coefficient.

#### SPM analysis

Voxel-wise statistical analyses were conducted using SPM12. Smoothed and spatially normalized SUVR images were entered into a one-way ANOVA model with the diagnostic group as the between-subject factor. Pairwise group comparisons (i.e., HC − vs. MCI+, HC − vs. AD, and MCI − vs. MCI+) were performed at the contrast specification stage within the SPM results interface. In addition, pairwise group comparisons (i.e., comparisons of the whole HC group vs. MCI+, as well as the whole HC group vs. AD) were also implemented (Online Resources 6 and 7). Statistical maps were thresholded at voxel-level uncorrected *p* < 0.001, with no cluster-level correction.

#### Regression analysis

To further explore the relationship between regional [^18^F]SMBT-1 SUVRs and cognitive functions, all study participants (*n* = 91) were included in the following analyses. We performed linear regression analyses adjusted for centiloid values, age, sex, and years of education. MMSE and ADAS-Jcog scores were used as cognitive outcomes. Standardized residuals were calculated for both predictors (regional SUVRs) and outcomes (cognitive scores) using the adjusted models, and partial correlation plots were constructed.

#### Mediation analysis

To elucidate the mechanistic pathway linking Aβ pathology, astrogliosis, and cognitive impairment, we conducted a mediation analysis using PROCESS macro (version 5.0) in SPSS (Andrew F. Hayes, www.processmacro.org) [31]. Model 4 (with 5,000 bootstrap resamples) was used to assess the indirect effect of the Aβ burden quantified in centiloid units (X) on cognitive performance (Y) via regional [^18^F]SMBT-1 SUVRs (M), controlling for age, sex, and education. The significance of the indirect effects was determined using percentile bootstrap confidence intervals, based on 5,000 resamples. A 95% confidence interval that did not include zero was considered to be indicative of a statistically significant mediation effect. In this analysis, global Aβ pathology (centiloid) was set as the independent variable (X).

## Results

### Demographic data

Table 1 summarizes the demographic and clinical characteristics of each subgroup, including HC−, HC+, MCI−, MCI+, and AD groups. Statistical analysis showed no significant differences in age or sex distribution across the subgroups. Across all subgroups, the number of female participants was relatively larger than that of male participants. The AD subgroup had significantly fewer years of education than the HC subgroup (*p* < 0.05).

**Table 1:** Demographics and cognitive test scores

Characteristics	HC−	HC+	MCI−	MCI+	AD
n	**27**	**8**	**19**	**25**	**12**
Age	**73.7 ± 7.2**	**78.8 ± 3.3**	**78.8 ± 5.9**	**78.0 ± 5.4**	**72.6 ± 14.1**
Sex					
Male	**10**	**3**	**5**	**9**	**4**
Female	**17**	**5**	**14**	**16**	**8**
Education(years)	**14.4 ± 2.5**	**12.1 ± 1.6**	**12.4 ± 2.4**	**13.4 ± 2.3**	**11.9 ± 2.6** ^*^
Cognitive Performance					
MMSE	**28.6 ± 1.5**	**28.6 ± 1.5**	**25.5 ± 2.6** ^**^	**26.0 ± 2.3** ^*^	**18.6 ± 6.5** ^***^
ADAS-Jcog^a^	**7.5 ± 3.1 (24)**	**11.2 ± 1.3 (5)**	**11.4 ± 4.3 (18)**	**14.1 ± 4.1 (18)**	**28.5 ± 16.2 (9)** ^***^
WMS-LMII	**19.6 ± 6.5**	**14.6 ± 7.8**	**7.9 ± 7.3** ^***^	**4.8 ± 7.1** ^***^	**0.3 ± 0.9** ^***^
Centiloid(Aβ)	**1.9 ± 9.7**	**65.9 ± 23.8** ^***^	**7.2 ± 9.8**	**75.6 ± 26.2** ^***^	**81.8 ± 19.3** ^***^

Data are presented as the mean ± standard deviation (SD) for continuous variables and n for categorical variables. Groups were compared using one-way ANOVA for continuous variables and chi-square test for categorical variables; overall p-values are shown in the rightmost column

ᵃ Numbers in parentheses indicate the number of participants with available ADAS-Jcog data

*HC-* healthy controls without amyloid, *HC+* healthy controls with amyloid, *MCI-* mild cognitive impairment without amyloid, *MCI+* mild cognitive impairment with amyloid, *AD* Alzheimer’s disease

Post-hoc comparisons versus the HC- group:

* p < 0.05

** p < 0.01

*** p < 0.001

Regarding cognitive performance, both the MCI and AD subgroups showed significantly lower scores than the HC group, indicating a progressive cognitive decline (*p* < 0.05). Additionally, participants in the HC+, MCI+, and AD subgroups had significantly higher Centiloid values than their Aβ-negative counterparts (HC− and MCI−), further confirming the presence of amyloid deposition (*p* < 0.001).

### ROI analysis

ROI analysis began with a foundational assessment of reference tissues. The raw SUV values within each of the four candidate reference regions (CGM, CWM, WC, and SWM) showed no significant differences among the clinical subgroups (Online Resources 2 and 3), confirming the stability of the reference tissues across the disease spectrum.

**Fig. 1:**
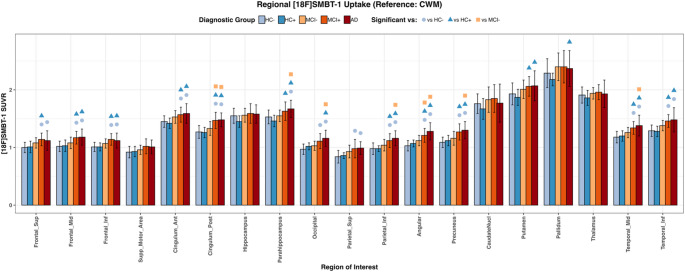
Group differences in regional brain uptake on [¹⁸F]SMBT-1 PET across the Alzheimer’s disease continuum. Mean standardized uptake value ratio (SUVR) in 19 regions of interest for each diagnostic group was calculated using cerebellar white matter (CWM) as the reference region. Representative 19 regions with highest statistical significances were selected from the whole-brain ROI analysis for clear visualization of the main findings, including 49 regions with statistical significance out of 95 regions. Error bars represent standard deviation (SD). Statistical significance from post-hoc tests is indicated by symbols placed above the corresponding bars. Significance symbols: A blue circle (●) indicates a significant difference compared to the HC− group; a green triangle (▲) indicates a significant difference compared to the HC+ group; and an orange square (■) indicates a significant difference compared to the MCI− group. Abbreviations: AD, Alzheimer’s disease; CWM, cerebellar white matter; Angular, Angular Gyrus; CaudateNucl, Caudate Nucleus; Cingulum_Ant, Anterior Cingulate Gyrus; Cingulum_Post, Posterior Cingulate Gyrus; Frontal_Inf, Inferior Frontal Gyrus; Frontal_Mid, Middle Frontal Gyrus; Frontal_Sup, Superior Frontal Gyrus; Parietal_Inf, Inferior Parietal Lobule; Parietal_Sup, Superior Parietal Lobule; Supp_Motor_Area, Supplementary Motor Area; Temporal_Inf, Inferior Temporal Gyrus; Temporal_Mid, Middle Temporal Gyrus

**Fig. 2:**
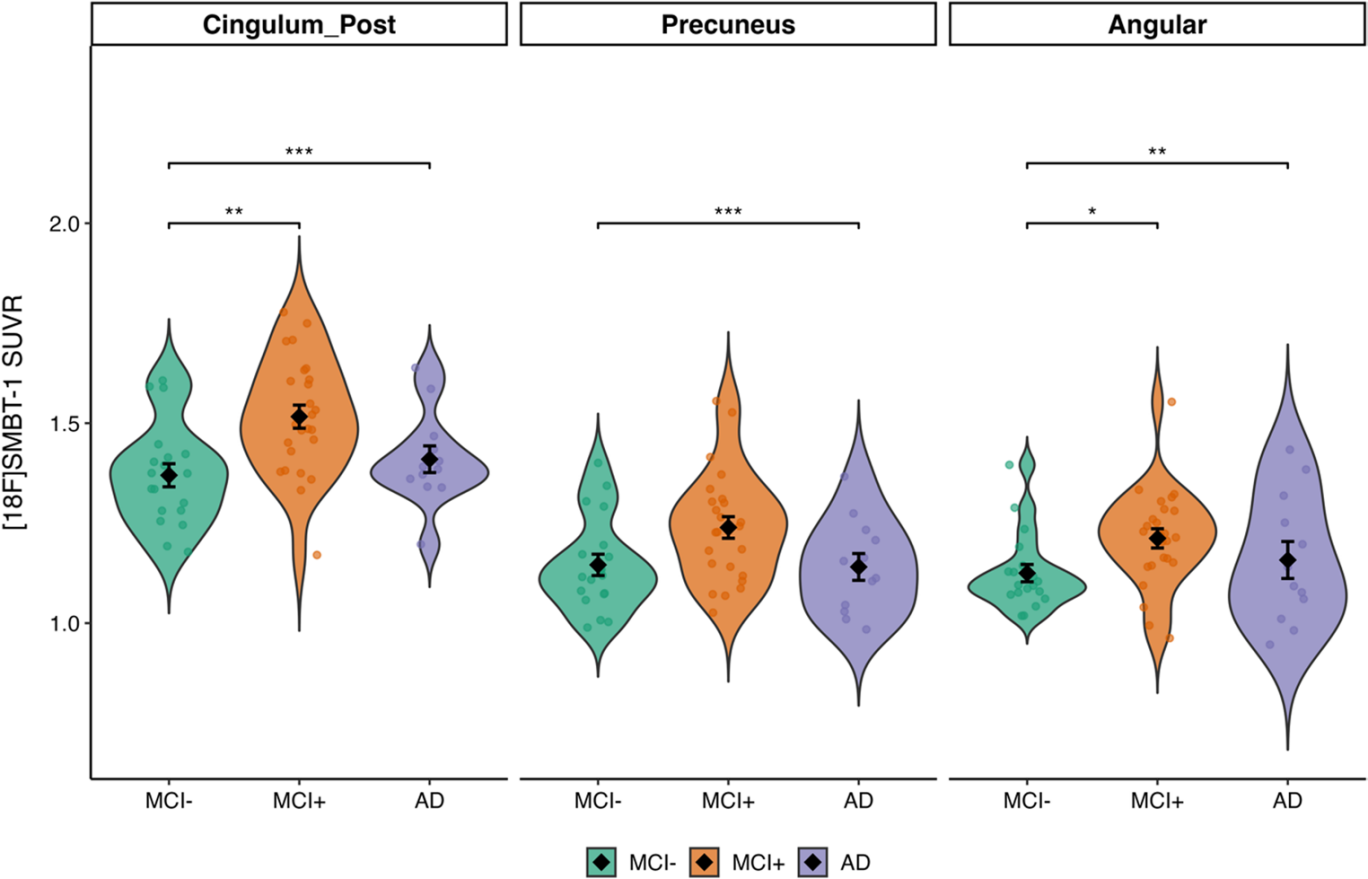
Regional differences in [^18^F]SMBT-1 SUVR in key posterior cortical regions. Quantitative region-of-interest analysis confirms the significant elevation of [^18^F] SMBT-1 SUVR in the MCI+ group compared to the MCI− group, with a progressive increase towards the AD group. Violin plots illustrate the data distribution, with the black diamond representing the group mean and error bars indicating the standard error of the mean (SEM). *p < 0.05, **p < 0.01, ***p < 0.001. Abbreviations: AD, Alzheimer’s disease; Angular, Angular Gyrus; Cingulum_Post, Posterior Cingulate Gyrus

Subsequently, SUVRs were calculated using these references, revealing both consistent and divergent patterns (Figs. 1 and 2; Table 2, and Online Resource 4). The three cerebellar-based references (CGM, CWM, and WC) yielded broadly consistent patterns. These methods all robustly showed that symptomatic, amyloid-positive groups (MCI+ and AD) had significantly higher SUVR than healthy controls (HC−) in widespread cortical areas, including the frontal gyri, posterior cingulate, and angular gyrus. It also showed that asymptomatic amyloid-positive individuals (HC+) had significantly lower uptake than symptomatic groups (MCI+ and AD), and amyloid-positive MCI (MCI+) subjects had significantly higher uptake than their amyloid-negative counterparts (MCI–) in posterior hubs, such as the posterior cingulate and angular gyrus, which are well-established early and highly vulnerable regions of the Alzheimer’s disease continuum [32, 33]. Among the above-mentioned three cerebellar references, CWM tended to show the most widespread areas of significance. In contrast, SWM-based SUVR quantification uniquely revealed significant group differences in several subcortical and brainstem structures such as the putamen, pallidum, and midbrain (Table 2). In addition, when comparing the entire HC group (HC– and HC+ combined) with the amyloid-positive MCI and AD groups, respectively, ROI-based analyses showed that both MCI+ and AD exhibited significantly elevated SMBT-1 SUVRs across almost all cortical regions compared to the whole HC group (HC– and HC+ combined)(Online Resource 5).

**Table 2:** Group comparisons of SUVR (MCI + vs. HC– and AD vs. HC–) across regions of interest and reference regions (p-values)

	Reference Regions								
	CGM		CWM		WC		SWM		
	MCI+	AD	MCI+	AD	MCI+	AD	MCI+	AD	
Frontal_Sup	**		***	*	***				
Frontal_Mid	***	*	***	***	***	*			
Frontal_Inf	***		***	**	***				
Supp_Motor_Area					*				
Cingulum_Ant			*	*					
Cingulum_Post	***	***	***	***	***	***	**		
Hippocampus							*	***	
Parahippocampus				*					
Occipital	**	**	***	****	***	***			
Parietal_Sup	**	*	**	*	***	*			
Parietal_Inf	**	**	***	***	***	***			
Angular	***	***	***	***	***	***	**	**	
Precuneus	***	***	***	***	***	***	*		
CaudateNuc									
Putamen								*	
Pallidum							*	**	
Thalamus							**	***	
Temporal_Mid	**	**	***	***	***	**			
Temporal_Inf	**		***	***	***	*			
Midbrain							***	***	
Pons							***	***	

This table shows the significant SUVR differences (*p* < 0.05) between MCI + vs. HC and AD vs. HC in the four reference regions. The vertical axis lists the [¹⁸F]SMBT-1 regions of interest (ROIs), and the horizontal axis shows the four reference regions (CGM, CWM, WC, and SWM) used for SUVR calculation. Representative 21 regions with highest statistical significances were selected from the whole-brain ROI analysis for clear visualization of the main findings, including 49 regions with statistical significance out of 95 regions

The blank cells indicate non-significant difference between the two groups (*p* ≥ 0.05). Significance levels are indicated as * *p* < 0.05, ** *p* < 0.01, and *** *p* < 0.001

*CGM* cerebellar grey matter, *CWM* cerebellar white matter, *WC* whole cerebellum, *SWM* subcortical white matter, *Angular* Angular Gyrus, *CaudateNucl* Caudate Nucleus, *Cingulum_Ant* Anterior Cingulate Gyrus, *Cingulum_Post* Posterior Cingulate Gyrus, *Frontal_Inf* Inferior Frontal Gyrus, *Frontal_Mid* Middle Frontal Gyrus, *Frontal_Sup* Superior Frontal Gyrus, *Parietal_Inf* Inferior Parietal Lobule, *Parietal_Sup* Superior Parietal Lobule, *Supp_Motor_Area* Supplementary Motor Area, *Temporal_Inf* Inferior Temporal Gyrus, *Temporal_Mid* Middle Temporal Gyrus

### White matter sub-analysis

**Fig. 3:**
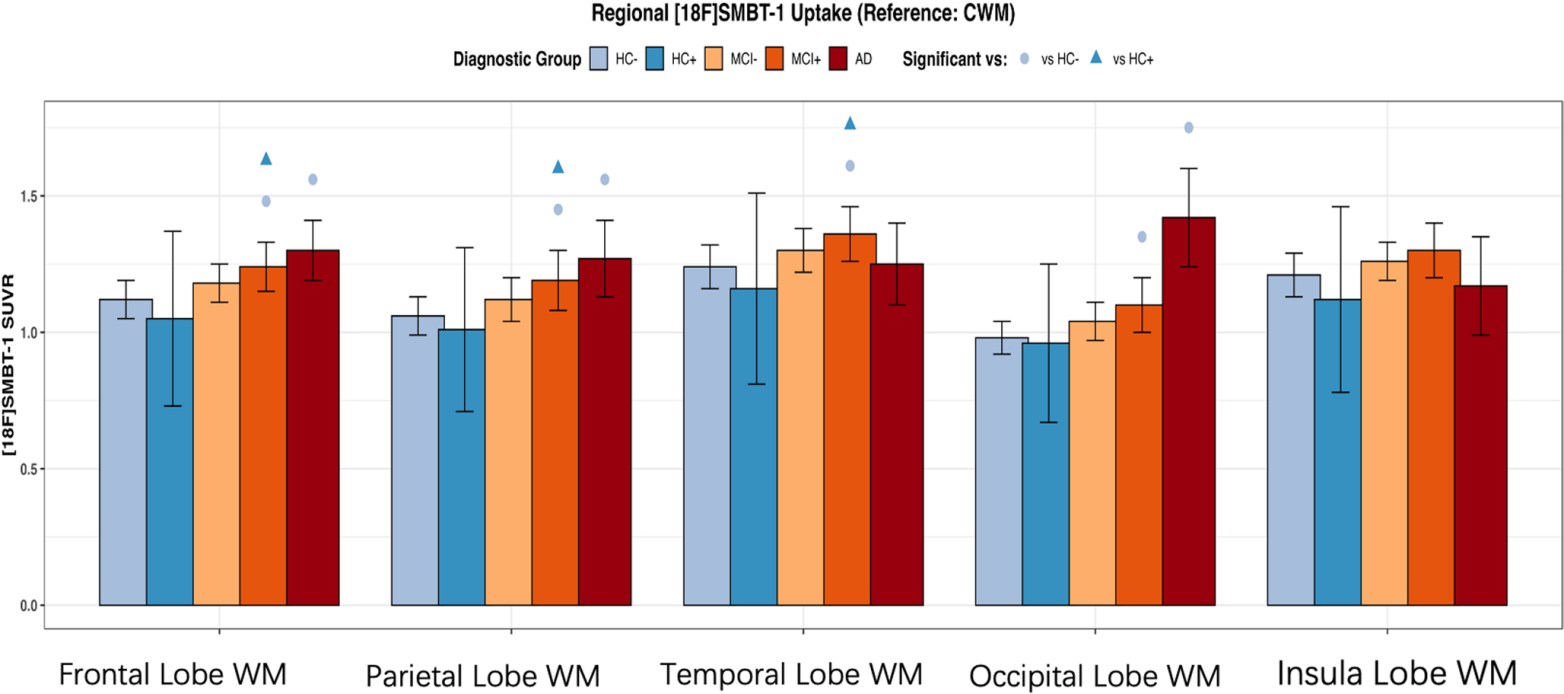
Group comparison of regional [¹⁸F]SMBT-1 SUVR in major lobar white matter regions (reference: CWM). Mean [¹⁸F]SMBT-1 standardized uptake value ratio (SUVR) values were calculated and presented for the five diagnostic groups. Error bars denote standard deviation. SUVRs were derived using the cerebellar white matter (CWM) as the reference region. Significance symbols: A blue circle (●) indicates a significant difference compared to the HC− group; and a green triangle (▲) indicates a significant difference compared to the HC+ group. Abbreviations: AD, Alzheimer’s disease; HC Aβ-, amyloid-β negative healthy controls; HC Aβ+, amyloid-β positive healthy controls; MCI Aβ-, amyloid-β negative mild cognitive impairment; MCI Aβ+, amyloid-β positive mild cognitive impairment; SUVR, Standardized Uptake Value Ratio; CWM, cerebellar white matter

Given the recent neuropathological evidence showing extensive MAO-B positive reactive astrogliosis in white matter regions [34], we performed an additional exploratory analysis of white matter SUVRs using the CWM reference (Fig. 3). Across the frontal, parietal, temporal, occipital, and insular white matter regions, both MCI+ and AD showed significantly higher [¹⁸F]SMBT-1 uptake than HC−, whereas no significant differences were observed between MCI+ and MCI−. In contrast, the insular white matter showed no significant group differences. These patterns support regional variability in white-matter astrogliosis.

### SPM analysis

**Fig. 4:**
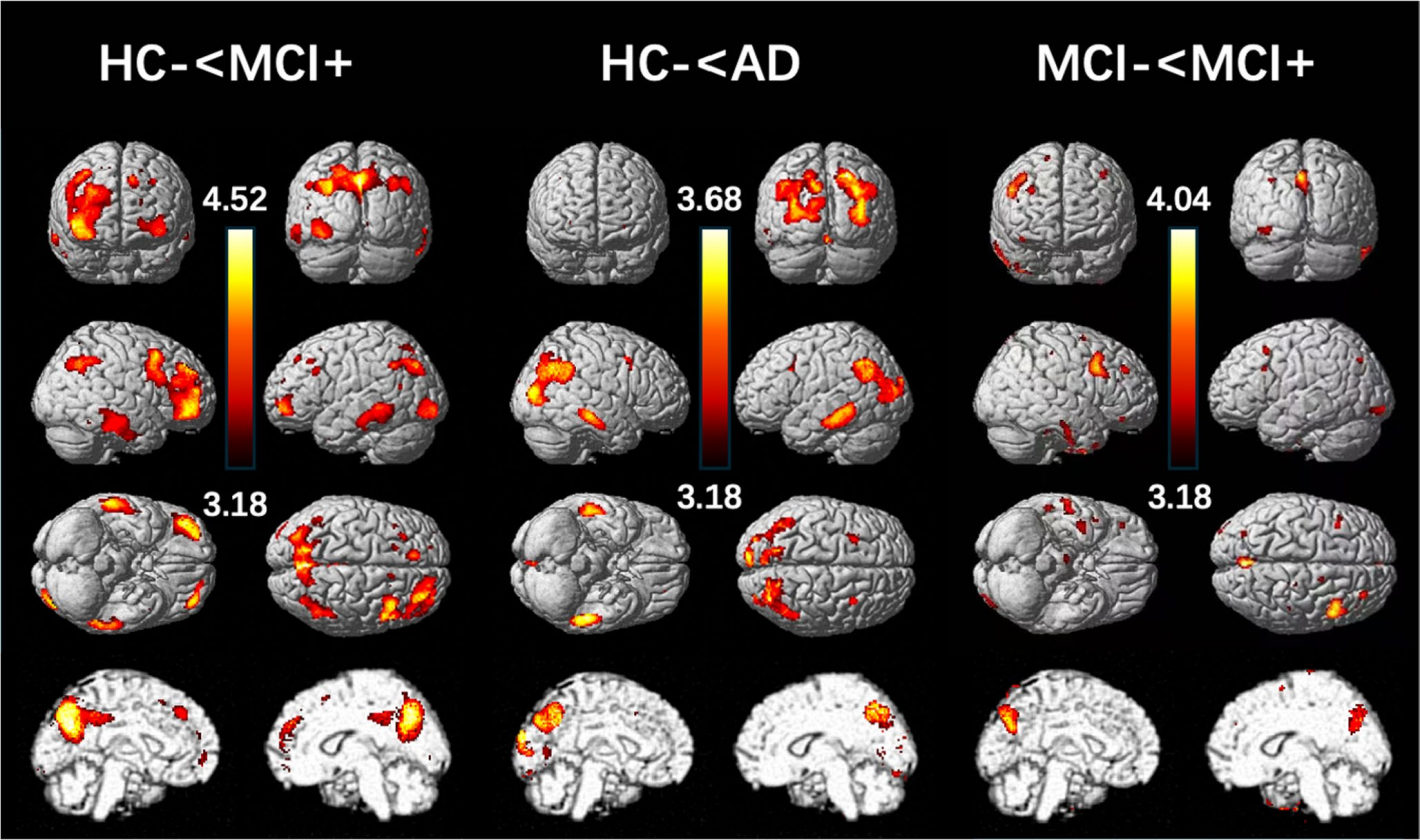
Voxel-wise group differences in [^18^F]SMBT-1 uptake. Statistical parametric maps on a standard brain template, showing regions of significantly increased [¹⁸F]SMBT-1 uptake for three comparisons: MCI+ vs. HC–, AD vs. HC–, and MCI+ vs. MCI–. Maps are displayed from top, lateral, and bottom orientations, with the color bar representing T-values Statistical threshold: *p* < 0.001, uncorrected, no cluster-extent threshold. Three-dimensional (3D) rendered images were generated by Statistical parametric mapping (SPM) software package, in which 2 types of 3D rendered images are combined in this figure. Surface-rendered images are default style of SPM12 (TOP three rows), and medial brain image is shown in SPM96-like style (BOTTOM), both generated by SPM12. Abbreviations: AD, Alzheimer’s disease; HC, amyloid-negative healthy controls; MCI, amyloid-negative mild cognitive impairment; MCI+, amyloid-positive mild cognitive impairment

In addition to the ROI-based analysis, voxel-wise comparisons using SUVR images normalized to CWM further support our findings. Compared with the HC− group, both the MCI + and AD groups showed significantly elevated [¹⁸F]SMBT-1 binding in widespread cortical areas, including the precuneus, angular gyrus, and middle temporal gyrus (Fig. 4 and Online Resource 6). These regions are known to be key hubs of the default mode network (DMN) and are among the earliest affected regions in Alzheimer’s pathology. Moreover, the MCI+ group exhibited a significantly higher SUVR than the MCI− group in the posterior cortical areas such as the precuneus. These patterns are in line with other PET studies on cognitive disorders, and reinforce the role of the posterior midline and parietal regions in amyloid-associated astrocyte reactivity.

In addition, comparisons of whole HC group (HC– and HC+ combined) with the MCI + and AD groups, respectively, these voxel-wise SPM maps demonstrated widespread cortical increases in [¹⁸F]SMBT-1 uptake for both HC vs. MCI + and HC vs. AD comparisons (Online Resources 6 and 7).

## The relationship between [^18^F]SMBT-1 distribution, cognitive performance, and CL

### Correlation analysis

To investigate the clinical and pathological relevance of regional astrogliosis, we examined the relationship among [^18^F]SMBT-1 binding, cognitive performance, and amyloid burden (Table 3). These analyses were performed across the entire study population (*n* = 91). Overall, elevated [^18^F]SMBT-1 binding was significantly associated with poor global cognitive function. Higher tracer uptake correlated with lower MMSE scores across widespread cortical regions, with the strongest associations found in the angular gyrus (*r* = −0.495), inferior parietal lobule (*r* = −0.447), and posterior cingulate (*r* = −0.411). Similarly, higher uptake in the frontal and temporal areas was correlated with poorer performance on the ADAS-Jcog. Notably, the most robust associations were observed for episodic memory performance (WMS-LMII). Significant negative correlations were found in key hubs of the DMN, including the posterior cingulate (*r* = −0.519), angular gyrus (*r* = −0.485), and precuneus (*r* = −0.485), indicating that greater astrogliosis in these regions is closely linked to memory decline.

**Table 3:** Correlation between regional [^18^F]SMBT-1 binding, cognitive performance, and centiloid

SMBT1 region	MMSE		ADAS-Jcog		WMS-LMII		Centiloid	
	*r*	*p*	*r*	*p*	*r*	*p*	*r*	*p*
Frontal_Sup	−0.351	0.001^**^	0.128	0.282	−0.296	0.004^**^	0.226	0.031^*^
Frontal_Mid	−0.328	0.002^**^	0.255	0.030^*^	−0.381	0.000^***^	0.491	0.000^***^
Frontal_Inf	−0.330	0.001^**^	0.245	0.037^*^	−0.365	0.000^***^	0.359	0.000^***^
Supp_Motor_Area	−0.200	0.057	0.172	0.146	−0.252	0.016^*^	0.309	0.003^**^
Insula	−0.284	0.006^**^	0.134	0.259	−0.363	0.000^***^	0.247	0.018^*^
Cingulum_Ant	−0.302	0.004^**^	0.182	0.124	−0.346	0.001^**^	0.245	0.019^*^
Cingulum_Post	−0.411	0.000^***^	0.368	0.001^**^	−0.519	0.000^***^	0.482	0.000^***^
Hippocampus	−0.048	0.649	−0.119	0.314	−0.116	0.275	−0.010	0.924
Parahippocampus	−0.276	0.008^**^	0.038	0.750	−0.386	0.000^***^	0.273	0.009^**^
Amygdala	−0.417	0.000^***^	0.285	0.014^*^	−0.450	0.000^***^	0.313	0.002^**^
Calcarine	−0.298	0.004^**^	0.233	0.047^*^	−0.425	0.000^***^	0.473	0.000^***^
Cuneus	−0.332	0.001^**^	0.260	0.026^*^	−0.402	0.000^***^	0.425	0.000^***^
Lingual	−0.258	0.014^*^	0.149	0.209	−0.401	0.000^***^	0.367	0.000^***^
Occipital	−0.375	0.000^***^	0.169	0.153	−0.367	0.000^***^	0.516	0.000^***^
Parietal_Sup	−0.345	0.001^**^	0.218	0.064	−0.343	0.001^**^	0.417	0.000^***^
Parietal_Inf	−0.447	0.000^***^	0.285	0.014^*^	−0.369	0.000^***^	0.424	0.000^***^
Supra_Marginal	−0.335	0.001^**^	0.158	0.182	−0.368	0.000^***^	0.324	0.002^**^
Angular	−0.495	0.000^***^	0.392	0.001^**^	−0.485	0.000^***^	0.470	0.000^***^
Precuneus	−0.396	0.000^***^	0.272	0.020^*^	−0.427	0.000^***^	0.457	0.000^***^
CaudateNucl	−0.066	0.533	−0.228	0.052	−0.197	0.062	0.015	0.888
Putamen	−0.342	0.001^**^	0.161	0.173	−0.314	0.002^**^	0.143	0.175
Pallidum	−0.208	0.048^*^	0.030	0.802	−0.143	0.176	0.034	0.752
Thalamus	−0.086	0.417	−0.124	0.297	−0.079	0.457	0.047	0.655
Temporal_Sup	−0.226	0.031^*^	−0.079	0.506	−0.304	0.003^**^	0.224	0.032^*^
Temporal_Mid	−0.341	0.001^**^	0.164	0.166	−0.437	0.000^***^	0.418	0.000^***^
Temporal_Inf	−0.365	0.000^***^	0.086	0.469	−0.414	0.000^***^	0.411	0.000^***^
Midbrain	−0.240	0.022^*^	0.177	0.135	−0.124	0.240	0.024	0.819
Pons	−0.226	0.031^*^	0.088	0.460	−0.114	0.280	0.068	0.519

Representative 28 regions with highest statistical significances were selected from the whole-brain ROI analysis showing meaningful correlations with the variables of interest, including 68 regions with statistical significance out of 95 regions

Bolded fonts Significant associations (*p* < 0.05)

*Angular* Angular Gyrus, *CaudateNucl* Caudate Nucleus, *Cingulum_Ant* Anterior Cingulate Gyrus, *Cingulum_Post* Posterior Cingulate Gyrus, *Frontal_Inf* Inferior Frontal Gyrus, *Frontal_Mid* Middle Frontal Gyrus, *Frontal_Sup* Superior Frontal Gyrus, *Parietal_Inf* Inferior Parietal Lobule, *Parietal_Sup* Superior Parietal Lobule, *Supp_Motor_Area* Supplementary Motor Area, *Temporal_Inf* Inferior Temporal Gyrus, *Temporal_Mid* Middle Temporal Gyrus

Finally, regional [^18^F]SMBT-1 binding was also significantly and positively correlated with amyloid burden (centiloid). The strongest relationships were observed in cortical association areas such as the occipital (*r* = 0.516) and middle frontal (*r* = 0.491) cortices. Taken together, these findings suggest that regional astrogliosis is closely linked not only to its upstream pathological driver (amyloid) but also to its downstream clinical consequences (cognitive and memory impairment).

### Adjusted regression analysis

**Fig. 5:**
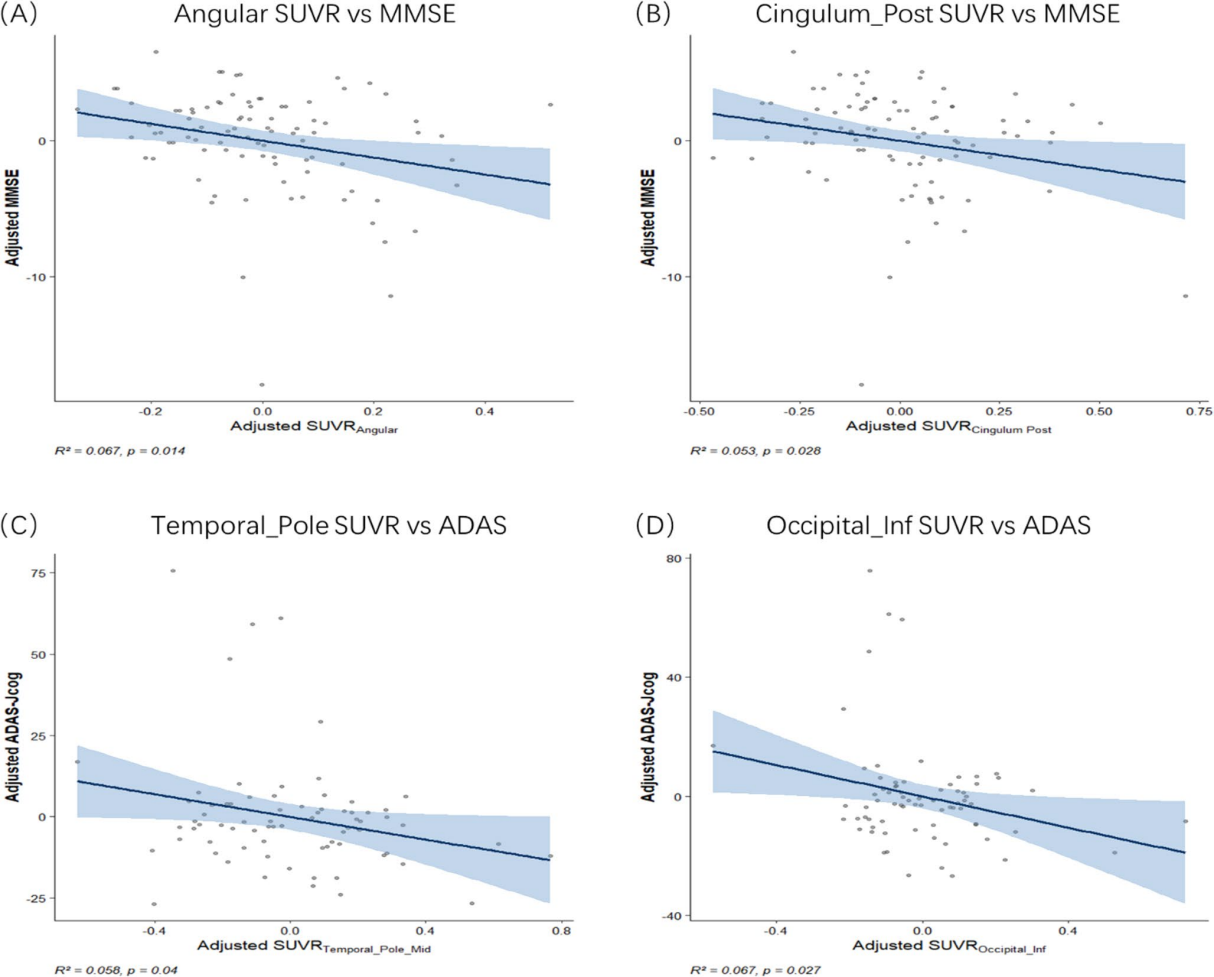
Partial regression plots showing the relationship between regional [^18^F]SMBT-1 SUVR and cognitive scores. (**A**–**B**) Higher SUVRs in the angular gyrus and posterior cingulate were associated with lower MMSE scores (**C**–**D**) Higher SUVRs in the temporal pole and inferior occipital gyrus correlated with poorer ADAS-cog performance. Plots display adjusted residuals with linear regression lines and 95% confidence intervals (CIs). R² and p values are shown for each association. Abbreviations: Angular, Angular Gyrus; Cingulum_Post, Posterior Cingulate Gyrus; Occipital_Inf, Inferior Occipital Gyrus

To account for confounders including centiloids, age, sex, and education, linear regression analyses were conducted. Figure 5 displays partial regression plots of the key regions. Notably, the adjusted SUVR in the angular gyrus and posterior cingulate was significantly negatively associated with MMSE (Angular: R² = 0.067, *p* = 0.014; Cingulum Post: R² = 0.053, *p* = 0.028). In contrast, adjusted SUVRs in the inferior occipital and temporal pole cortices were positively associated with ADAS-Jcog (Occipital Inf: R² = 0.067, *p* = 0.027; Temporal Pole: R² = 0.058, *p* = 0.04), indicating that greater tracer uptake was correlated with worse cognitive scores. No significant regression was observed in the other regions.

### Mediation analysis

**Fig. 6:**
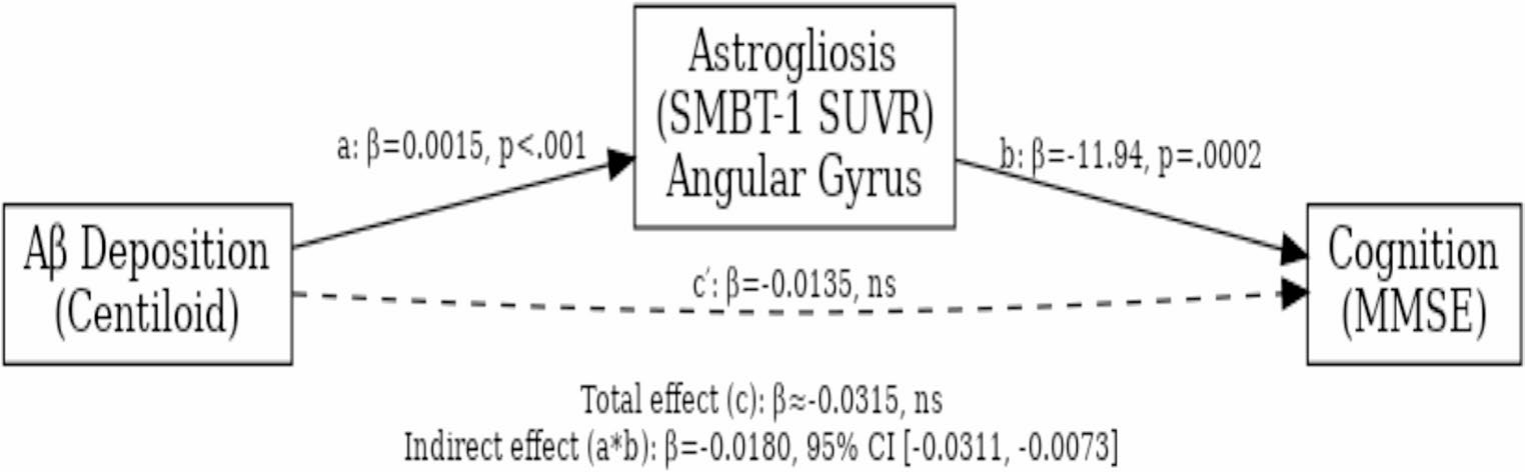
Mediation model of angular gyrus astrogliosis between Aβ deposition and cognition. A path analysis model illustrating the mediating role of astrogliosis in the angular gyrus astrogliosis ([¹⁸F]SMBT-1 SUVR) in the relationship between cerebral Aβ deposition (centiloids) and cognitive function (MMSE). Values on paths are unstandardized regression coefficients with corresponding p-values

Astrogliosis in the angular gyrus ([^18^F]SMBT-1 SUVR), a region showing robust associations with cognitive performance, was the mediator (M) and cognitive performance (MMSE score) was the dependent variable (Y). The analyses were adjusted for age, sex, and years of education. The detailed coefficients are listed in Table 4 and the mediation model is shown in Fig. 6.

**Table 4:** Mediation analysis of the effect of Aβ pathology on cognition through astrogliosis in the angular gyrus

Path (X = Aβ, M=Angular SMBT-1, Y=MMSE)	Effect (β)	SE	t	*p*	95% Confidence Interval
Indirect Effect Pathway					
Aβ → Astrogliosis (Path a)	0.0015	0.0003	4.533	< 0.001	[0.0008, 0.0022]
Astrogliosis → Cognition (Path b)	−11.9362	3.1213	−3.824	0.0002	[−18.1422, −5.7303]
Direct & Total Effects					
Aβ → Cognition (Direct Effect, c’)	−0.0135	0.0107	−1.257	0.212	[−0.0348, 0.0078]
Summary of Indirect Effect	Effect	Boot SE			Boot 95% Confidence Interval
Aβ → Astrogliosis → Cognition	−0.0180	0.0061			[−0.0311, −0.0073]

Results from ordinary least squares path analysis. The model tested the indirect effect of global Aβ pathology (X; centiloid) on cognitive performance (Y; MMSE score) through astrogliosis in the angular gyrus (M; [^18^F] SMBT-1 SUVR) while controlling for age, sex, and years of education. β is the unstandardized regression coefficient. The significance of the indirect effect was determined using a bootstrapping procedure with 5,000 samples, and a 95% confidence interval not containing zero was considered to be statistically significant. Abbreviations: *SE* Standard Error, *CI* Confidence Interval, *Boot* Bootstrap

Aβ pathology was a significant positive predictor of astrogliosis in the angular gyrus (Path a: β = 0.0015, *p* < 0.001). Astrogliosis, in turn, was a significant negative predictor of MMSE score (path b: β = −11.9362, *p* = 0.0002). The indirect effect was significant (effect = −0.0180, 95% CI [−0.0311, −0.0073]), indicating that a higher Aβ burden was associated with greater astrogliosis, which in turn was related to poorer cognitive performance. The direct effect of Aβ pathology on MMSE scores became non-significant after including the mediator (Path c′: β = −0.0135, *p* = 0.212). A significant but weaker mediation effect was also observed in the posterior cingulate cortex (Path a: β = 0.0028, *p* < 0.001), whereas mediation effects in frontal and occipital regions did not reach statistical significance.

## Discussion

Our study characterized in vivo reactive astrogliosis across the AD continuum—here defined as the amyloid-positive groups (HC+, MCI+, and AD) [35]—and contrasted these findings with key amyloid-negative comparison groups (HC− and MCI−). The central contribution of this study is the characterization of distinct astrogliosis patterns that differentiate these subgroups based on both amyloid status and clinical symptoms. And supplementary analyses combining the two HC subgroups (HC− and HC+) confirmed that the whole HC cohort exhibited significantly lower [^18^F]SMBT-1 uptake than the symptomatic MCI+ and AD groups (Online Resources 5, 6, and 7), indicating higher astrocyte reactivity in amyloid-positive symptomatic stages. Our analyses revealed that astrogliosis is not only elevated in symptomatic AD continuum groups (MCI+ and AD) relative to healthy controls (HC-), but also that its patterns distinguish between MCI with and without underlying amyloid pathology (MCI+ vs. MCI−). Furthermore, SMBT-1 uptake was significantly higher in symptomatic amyloid-positive (MCI+) individuals than in asymptomatic amyloid-positive (HC+) individuals. Together, these findings support the utility of [¹⁸F]SMBT-1 PET for characterizing astrocyte reactivity across biologically defined subgroups and delineating the heterogeneity of MCI.

Although the clinical AD diagnoses in this study followed the NIA-AA 2011 criteria, the conceptual framework used to define the AD continuum was updated to align with the 2024 NIA-AA biological research framework for biological interpretation of the imaging findings, in which Aβ positivity defines AD pathophysiology.

This pathological divergence has several clinical implications. In contrast to earlier reports with limited MCI samples [18], our well-powered cohort allowed for detailed examination of MCI subtypes. The AD-like pattern of astrogliosis in the MCI+ group strongly supports its classification as prodromal AD, which carries a much higher risk of progression to dementia [36, 37]. Conversely, the amyloid-negative MCI group aligns with a suspected non-Alzheimer’s pathophysiology (SNAP) profile often characterized by non-amyloid pathologies [38]. The SMBT-1 uptake pattern in the MCI− group, distinct from the AD-like pattern seen in MCI+, may partly reflect contributions from non-Aβ etiologies such as primary age-related tauopathy (PART) [39] or limbic-predominant age-related TDP-43 encephalopathy (LATE) [40], both of which preferentially affect medial temporal and limbic structures and arise independently of amyloid. Importantly, [¹⁸F]SMBT-1 reflects MAO-B-related astrogliosis rather than pathology-specific signatures, and therefore cannot distinguish PART or LATE from amyloid-positive MCI based on uptake alone. The divergent patterns between MCI− and MCI+ provide indirect support for pathological heterogeneity within the SNAP spectrum, whereas confirmation of disease-specific astrocytic profiles requires multimodal or longitudinal biomarker studies. The highest SUVR values were frequently observed in the MCI+ subgroup. This finding further supports the hypothesis that reactive astrogliosis may be a dynamic event, with its intensity potentially showing a nonlinear change across disease stages. This phenomenon appears to be in line with earlier autopsy findings [41] and suggests a potential spatiotemporal coupling between astrocyte reactivity and early Aβ deposition [3, 42], although the heterogeneous characteristics of astrogliosis in the early stages of non-AD pathologies should be studied in further details. Consistent with recent MAO-B histopathology, our exploratory white matter analysis showed elevated SMBT-1 uptake mainly in amyloid-positive symptomatic groups, although this pattern did not distinguish MCI+ from MCI−.

Beyond our group-level findings, our results with [¹⁸F]SMBT-1 add to the growing body of literature using PET to visualize reactive astrogliosis. As a tracer for MAO-B, [¹⁸F]SMBT-1 shares its biological target with the well-established tracer [¹¹C]-L-deprenyl ([¹¹C]DED). Notably, our observation of peak SUVR values in the MCI+ group, suggesting a nonlinear progression of astrogliosis, is highly consistent with previous in vivo studies using [¹¹C]DED, which also reported that MAO-B expression may not increase linearly with disease severity [8]. Our study, with its large and well-stratified MCI cohort, provides robust in vivo support for this dynamic pattern in the prodromal phase of AD. In contrast, other tracers have been developed to target alternative markers such as I2BS targeted by [¹¹C]BU99008 [15]. Although these tracers also show increased signals in AD, future comparative studies are needed to understand whether different biological markers of reactive astrocytes reveal distinct spatiotemporal patterns.

To date, we have investigated the relationship between regional astrogliosis and amyloid burden in order to better understand the pathological basis of these findings. Regional [^18^F]SMBT-1 binding was significantly and positively correlated with the centiloid values (Table 3), indicating a close association between astrocytic MAO-B binding and the degree of amyloid pathology [43–46]. In addition, the cognitive levels demonstrated by the MMSE, ADAS-Jcog, and WMS-LMII were significantly correlated with the uptake of [^18^F]SMBT-1 PET (Table 3; Fig. 5), supporting the relevance of astrocyte reactivity to clinical manifestations. [^18^F]SMBT-1 uptake was strongly correlated with centiloid values (Aβ burden) in the angular gyrus (*r* = 0.470), posterior cingulate (*r* = 0.482), occipital (*r* = 0.516), and middle frontal cortices (*r* = 0.491)(Table 3). This pattern suggests a crucial convergence of pathological and clinical relevance in the DMN core hubs. The angular gyrus was selected as the mediator because it is a vital integrative center that is strongly associated with both the upstream Aβ driver and downstream cognitive measures. This spatial overlap justifies its role as a key mechanistic mediator of the Aβ-to-cognition pathway.

The positive association between SMBT-1 uptake in the temporal pole and ADAS-Jcog performance suggests that astrocyte reactivity in this region reflects the early neurodegenerative processes relevant to cognitive decline. The temporal pole and adjacent medial temporal structures represent the earliest cortical sites of tau accumulation, as established by classical neuropathological staging [47] and supported by tau-PET studies that demonstrated initial tangle formation in these medial temporal regions [48]. Tau deposition in these temporal regions is closely associated with hippocampal atrophy in preclinical individuals, with baseline entorhinal and temporal pole tau predicting subsequent medial temporal thinning [49].

Importantly, recent multimodal biomarker evidence indicates that astrocyte reactivity modulates and amplifies the effects of Aβ on early tau phosphorylation. Aβ is associated with elevated phosphorylated tau only in individuals with high astrocyte reactivity, and this astrogliosis positive (Ast+) subgroup exhibits an AD-like spatial pattern of tau accumulation cross-sectionally and longitudinally [50].

Taken together, these findings suggest that increased SMBT-1 uptake in the temporal pole reflects reactive astrogliosis participating in early Aβ-associated tau processes, which may contribute to the decline in language and memory functions measured by ADAS-Jcog.

Building on this, our mediation analysis demonstrated that astrogliosis in the angular gyrus was associated with both Aβ pathology and cognitive performance. This finding provides quantitative evidence that astrogliosis may not be a mere bystander. Instead, our results may support the view that reactive astrogliosis could act as a key mechanistic link through which upstream Aβ toxicity is related to downstream neuronal dysfunction and cognitive impairment. A key strength of our study is the large, well-characterized MCI cohort. Furthermore, our methodological choices were based on a systematic comparative analysis to ensure optimal sensitivity [17, 20, 21, 51, 52].

This study has the following limitations. First, its cross-sectional design precludes definitive conclusions regarding cause-and-effect relationships. Second, “MCI−” designation can represent a heterogeneous population, potentially including the early stages of non-AD tauopathies [53]. Third, although standardized protocols were implemented, the use of multiple scanners should be acknowledged as a potential limitation. Fourth, PET signal measurements in atrophied brain regions may be susceptible to partial volume effects (PVE). Since PET acquisition was performed using static frames, SUVR measurements may also be influenced by neurodegenerative changes, such as reduced blood flow, loss of neurons and MAO-B expressing astrocytes, which may partially account for the lower SUV/SUVR values observed in AD patients compared with MCI+ subjects. Such PVE might also affect white matter findings, since higher signals in neighboring grey matter regions might “spill-in” into white matter ROIs, leading to increased SUV. These effects can be partially corrected by specific algorithms for partial volume correction (PVC), and can also be reduced by using a brain-dedicated PET scanner with a higher spatial resolution of 2 mm. Fifth, although the overall cohort size was substantial, the modest number of individuals in AD group (*n* = 12) resulted in limited statistical power, associated with lack of corrections for multiple comparisons in SPM and correlation analyses as well as limited generalizability of complex analyses such as mediation modeling. Finally, our cohort was not strictly controlled: a higher proportion of female participants and exceptional enrollment of an MCI subject with early urinary bladder cancer without symptoms 11 months after endoscopic operation, according to our inclusion criteria (Online Resource 1). Further multimodal longitudinal studies are required to dynamically track the interplay between these pathologies.

## Conclusion

In conclusion, this study provides in vivo evidence that reactive astrogliosis differs across biologically defined subgroups within a heterogeneous MCI landscape. Our findings indicate that [¹⁸F]SMBT-1 PET can characterize astrocytic differences between MCI subtypes and offer insights into the involvement of astrocytes within the Alzheimer’s disease continuum without implying a strictly linear relationship with clinical severity.

Rather than defining disease progression, our results highlight that SMBT-1 uptake is elevated in amyloid-positive MCI and AD, supporting its relevance to amyloid-associated astrocyte reactivity, particularly in prodromal AD (MCI+). By emphasizing the importance of reference region selection, we highlight that cerebellar references, especially the CWM, offer superior sensitivity in stratifying subgroups of the AD continuum, compared to other references such as SWM. These findings motivate future longitudinal and multimodal studies to further clarify the temporal trajectory of astrogliosis and explore the potential clinical utility of [¹⁸F]SMBT-1 for characterizing the early symptomatic stages of Alzheimer’s disease.

## Supplementary Information

Below is the link to the electronic supplementary material.

Supplementary Material 1 (DOCX 1.64 MB)

## Data Availability

The data that support the findings of this study are currently not publicly available because of privacy and ethical restrictions, as they contain information that could compromise the privacy of the research participants.

## References

[1] Masters CL. Neuropathology of Alzheimer’s disease. In: Burns A, O’Brien J, Ames D, editors. Dementia. 3rd ed. London: Hodder Arnold; 2005. pp. 393–407.

[2] Verkhratsky A, Nedergaard M. Physiology of astroglia. Physiol Rev. 2018;98(1):239–389. 10.1152/physrev.00042.2016. 29351512 10.1152/physrev.00042.2016 PMC6050349

[3] Verkhratsky A, Olabarria M, Noristani HN, Yeh CY, Rodríguez JJ. Astrocytes in Alzheimer’s disease. Neurotherapeutics. 2010;7(4):399–412. 10.1016/j.nurt.2010.05.017. 20880504 10.1016/j.nurt.2010.05.017 PMC5084302

[4] Beyer L, Stöcker H, Rujescu D, Holleczek B, Stockmann J, Nabers A, et al. Amyloid-β misfolding and GFAP predict risk of clinical Alzheimer’s disease diagnosis within 17 years. Alzheimers Dement. 2023;19(3):1020–8. 10.1002/alz.12745. 35852967 10.1002/alz.12745

[5] Cai Z, Wan CQ, Liu Z. Astrocyte and Alzheimer’s disease. J Neurol. 2017;264:2068–74. 10.1007/s00415-017-8593-x. 28821953 10.1007/s00415-017-8593-x

[6] Marutle A, Gillberg P-G, Bergfors A, Yu W, Ni R, Nennesmo I, et al. ³H-deprenyl and ³H-PIB autoradiography show different laminar distributions of astroglia and fibrillar β-amyloid in Alzheimer brain. J Neuroinflammation. 2013;10:90. 10.1186/1742-2094-10-90. 23880036 10.1186/1742-2094-10-90 PMC3733895

[7] Ni R, Röjdner J, Voytenko L, Dyrks T, Thiele A, Marutle A, et al. In vitro characterization of the regional binding distribution of amyloid PET tracer florbetaben and the glia tracers deprenyl and PK11195 in autopsy Alzheimer’s brain tissue. J Alzheimers Dis. 2021;80(4):1723–37. 10.3233/JAD-201344. 33749648 10.3233/JAD-201344 PMC8150513

[8] Carter SF, Schöll M, Almkvist O, Wall A, Engler H, Långström B, et al. Evidence for astrocytosis in prodromal Alzheimer disease provided by 11 C-deuterium-L-deprenyl: a multitracer PET paradigm combining 11 C-Pittsburgh compound B and 18F-FDG. J Nucl Med. 2012;53(1):37–46. 10.2967/jnumed.110.087031. 22213821 10.2967/jnumed.110.087031

[9] Rodriguez-Vieitez E, Ni R, Gulyás B, Tóth M, Häggkvist J, Halldin C, et al. Astrocytosis precedes amyloid plaque deposition in Alzheimer APPswe transgenic mouse brain: a correlative positron emission tomography and in vitro imaging study. Eur J Nucl Med Mol Imaging. 2015;42(7):1119–32. 10.1007/s00259-015-3047-0. 25893384 10.1007/s00259-015-3047-0 PMC4424277

[10] Schedin-Weiss S, Inoue M, Hromádková L, Teranishi Y, Yamamoto NG, Wiehager B, et al. Monoamine oxidase B is elevated in Alzheimer disease neurons, is associated with γ-secretase and regulates neuronal amyloid β-peptide levels. Alzheimers Res Ther. 2017;9(1):57. 10.1186/s13195-017-0279-1. 28764767 10.1186/s13195-017-0279-1 PMC5540560

[11] Smit T, Deshayes NAC, Borchelt DR, Kamphuis W, Middeldorp J, Hol EM. Reactive astrocytes as treatment targets in Alzheimer’s disease — systematic review of studies using the APPswePS1dE9 mouse model. Glia. 2021;69(8):1852–81. 10.1002/glia.23981. 33634529 10.1002/glia.23981 PMC8247905

[12] Ballweg A, Klaus C, Vogler L, Katzdobler S, Wind K, Zatcepin A, et al. [^18^F]F-DED PET imaging of reactive astrogliosis in neurodegenerative diseases: preclinical proof of concept and first-in-human data. J Neuroinflammation. 2023;20(1):68. 10.1186/s12974-023-02749-2. 36906584 10.1186/s12974-023-02749-2 PMC10007845

[13] Olsen M, Aguilar X, Sehlin D, Fang XT, Antoni G, Erlandsson A, et al. Astroglial responses to amyloid-beta progression in a mouse model of Alzheimer’s disease. Mol Imaging Biol. 2018;20(4):605–14. 10.1007/s11307-017-1153-z. 29297157 10.1007/s11307-017-1153-z

[14] Drake LR, Brooks AF, Mufarreh AJ, Pham JM, Koeppe RA, Shao X, et al. Deuterium kinetic isotope effect studies of a potential in vivo metabolic trapping agent for monoamine oxidase B. ACS Chem Neurosci. 2018;9(12):3024–7. 10.1021/acschemneuro.8b00219. 30074755 10.1021/acschemneuro.8b00219 PMC7589526

[15] Tyacke RJ, Myers JFM, Venkataraman A, Mick I, Turton S, Passchier J, et al. Evaluation of [¹C]BU99008, a PET ligand for the imidazoline₂ binding site in human brain. J Nucl Med. 2018;59(10):1597–602. 10.2967/jnumed.118.208009. 29523627 10.2967/jnumed.118.208009

[16] Ekblom J, Jossan SS, Bergström M, Oreland L, Walum E, Aquilonius SM. Monoamine oxidase B in astrocytes. Glia. 1993;8(2):122–32. 10.1002/glia.440080208. 8406673 10.1002/glia.440080208

[17] Villemagne VL, Harada R, Doré V, Furumoto S, Mulligan R, Kudo Y, et al. First-in-humans evaluation of ^18^F-SMBT-1, a novel ^18^F-labeled monoamine oxidase-B PET tracer for imaging reactive astrogliosis. J Nucl Med. 2022;63(10):1551–9. 10.2967/jnumed.121.263254. 35086898 10.2967/jnumed.121.263254 PMC9536703

[18] Villemagne VL, Harada R, Doré V, Furumoto S, Mulligan R, Kudo Y, et al. Assessing reactive astrogliosis with 18F-SMBT-1 across the Alzheimer’s disease spectrum. J Nucl Med. 2022;63(10):1560–9. 10.2967/jnumed.121.263255. 35086892 10.2967/jnumed.121.263255 PMC9536709

[19] Jack CR Jr, Andrews JS, Beach TG, Buracchio T, Dunn B, Graf A, et al. Revised criteria for diagnosis and staging of Alzheimer’s disease. Alzheimers Dement. 2024;20(8):5143–69. 10.1002/alz.13859. 38934362 10.1002/alz.13859 PMC11350039

[20] Lopresti BJ, Stehouwer J, Reese AC, Mason NS, Royse SK, Narendran R, et al. Kinetic modeling of the monoamine oxidase-B radioligand [^18^F]SMBT-1 in human brain with positron emission tomography. J Cereb Blood Flow Metab. 2024;44(11):1262–76. 10.1177/0271678X241254679. 38735059 10.1177/0271678X241254679 PMC11542143

[21] Hiraoka K, Mesfin B, Wu Y, Shimizu Y, Kikuchi A, Harada R, et al. Kinetic and quantitative analysis of [18F]SMBT-1 PET imaging for monoamine oxidase B. Ann Nucl Med. 2025. 10.1007/s12149-025-02083-y. 10.1007/s12149-025-02083-y PMC12559039 40663226

[22] Petersen RC. Mild cognitive impairment as a diagnostic entity. J Intern Med. 2004;256(3):183–94. 10.1111/j.1365-2796.2004.01388.x. 15324362 10.1111/j.1365-2796.2004.01388.x

[23] McKhann GM, Knopman DS, Chertkow H, Hyman BT, Jack CR, Kawas CH, et al. The diagnosis of dementia due to Alzheimer’s disease: recommendations from the National Institute on Aging-Alzheimer’s Association workgroups on diagnostic guidelines for Alzheimer’s disease. Alzheimers Dement. 2011;7(3):263–9. 10.1016/j.jalz.2011.03.005. 21514250 10.1016/j.jalz.2011.03.005 PMC3312024

[24] Harada R, Hayakawa Y, Ezura M, Lerdsirisuk P, Du Y, Ishikawa Y, et al. ^18^F-SMBT-1: a selective and reversible PET tracer for monoamine oxidase-B imaging. J Nucl Med. 2021;62(2):253–8. 10.2967/jnumed.120.244400. 32646880 10.2967/jnumed.120.244400

[25] Harada R, Shimizu Y, Du Y, Ishikawa Y, Iwata R, Kudo Y, et al. The Role of Chirality of [^18^F]SMBT-1 in Imaging of Monoamine Oxidase-B. ACS Chem Neurosci. 2022;13(3):322–9. 10.1021/acschemneuro.1c00655. 35049267 10.1021/acschemneuro.1c00655

[26] Klunk WE, Koeppe RA, Price JC, Benzinger TL, Devous MD, Sr, Jagust WJ, et al. The Centiloid Project: standardizing quantitative amyloid plaque estimation by PET. Alzheimers Dement. 2015;11(1):1–e154. 10.1016/j.jalz.2014.07.003. 25443857 10.1016/j.jalz.2014.07.003 PMC4300247

[27] Battle MR, Pillay LC, Lowe VJ, Knopman D, Kemp B, Rowe CC, et al. Centiloid scaling for quantification of brain amyloid with [18F]flutemetamol using multiple processing methods. EJNMMI Res. 2018;8:107. 10.1186/s13550-018-0456-7. 30519791 10.1186/s13550-018-0456-7 PMC6281542

[28] PMOD Technologies Ltd. Centiloid Analysis. Version 3.9. Zurich. Switzerland: PMOD Technologies Ltd.; 2019.

[29] Collij LE, Bollack A, La Joie R, Shekari M, Bullich S, Roé-Vellvé N, et al. Centiloid recommendations for clinical context-of-use from the AMYPAD consortium. Alzheimers Dement. 2024;20(12):9037–48. 10.1002/alz.14336. 39564918 10.1002/alz.14336 PMC11667534

[30] Yamane T, Ishii K, Sakata M, Ikari Y, Nishio T, Ishii K, et al. Inter-rater variability of visual interpretation and comparison with quantitative evaluation of (11)C-PiB PET amyloid images of the Japanese Alzheimer’s Disease Neuroimaging Initiative (J-ADNI) multicenter study. Eur J Nucl Med Mol Imaging. 2017;44(5):850–7. 10.1007/s00259-016-3591-2. 27966045 10.1007/s00259-016-3591-2

[31] Hayes AF. Introduction to mediation, moderation, and conditional process analysis: a regression-based approach. 3rd ed. New York: The Guilford Press; 2022.

[32] Ishii K. Pet approaches for diagnosis of dementia. AJNR Am J Neuroradiol. 2014;35(11):2030–8. 10.3174/ajnr.A3695. 23945233 10.3174/ajnr.A3695 PMC7965187

[33] Marcus C, Mena E, Subramaniam RM. Brain PET in the diagnosis of Alzheimer’s disease. Clin Nucl Med. 2014;39(10):e413–26. 10.1097/RLU.0000000000000547. 25199063 10.1097/RLU.0000000000000547 PMC4332800

[34] Jaisa-Aad M, Muñoz-Castro C, Healey MA, Hyman BT, Serrano-Pozo A. Characterization of monoamine oxidase-B (MAO-B) as a biomarker of reactive astrogliosis in Alzheimer’s disease and related dementias. Acta Neuropathol. 2024;147(1):66. 10.1007/s00401-024-02712-2. 38568475 10.1007/s00401-024-02712-2 PMC10991006

[35] Jack CR Jr, Bennett DA, Blennow K, Carrillo MC, Dunn B, Haebelein SB, et al. NIA-AA research framework: toward a biological definition of Alzheimer’s disease. Alzheimers Dement. 2018;14(4):535–62. 10.1016/j.jalz.2018.02.018. 29653606 10.1016/j.jalz.2018.02.018 PMC5958625

[36] Chapleau M, Iaccarino L, Soleimani-Meigooni D, Rabinovici GD. The role of amyloid PET in imaging neurodegenerative disorders: a review. J Nucl Med. 2022;63(Suppl 1):S13-9. 10.2967/jnumed.121.263233. 10.2967/jnumed.121.263195 PMC9165727 35649652

[37] Johnson KA, Fox NC, Sperling RA, Klunk WE. Brain imaging in Alzheimer disease. Cold Spring Harb Perspect Med. 2012;2(4):a006213. 10.1101/cshperspect.a006213. 22474610 10.1101/cshperspect.a006213 PMC3312396

[38] Wisse LEM, Butala N, Das SR, Davatzikos C, Dickerson BC, Vaishnavi SN, et al. Alzheimer’s Disease Neuroimaging Initiative. Suspected non-AD pathology in Mild Cognitive Impairment. Neurobiol Aging. 2015;36(12):3152–62. 10.1016/j.neurobiolaging.2015.08.027. 26422359 10.1016/j.neurobiolaging.2015.08.029 PMC4641774

[39] Crary JF, Trojanowski JQ, Schneider JA, Abisambra JF, Abner EL, Alafuzoff I, et al. Primary age-related tauopathy (PART). Acta Neuropathol. 2014;128(6):755–66. 10.1007/s00401-014-1349-0. 25348064 10.1007/s00401-014-1349-0 PMC4257842

[40] Nelson PT, Dickson DW, Trojanowski JQ, Jack CR Jr, Boyle PA, Arfanakis K, et al. Limbic-predominant age-related TDP-43 encephalopathy (LATE): consensus working group report. Brain. 2019;142(6):1503–27. 10.1093/brain/awz099. 31039256 10.1093/brain/awz099 PMC6536849

[41] Gulyás B, Pavlova E, Kása P, et al. Activated MAO-B in the brain of Alzheimer patients, demonstrated by [11C]-L-deprenyl using whole hemisphere autoradiography. Neurochem Int. 2011;58(1):60–8. 10.1016/j.neuint.2010.10.013. 21075154 10.1016/j.neuint.2010.10.013

[42] Lichtenstein MP, Carriba P, Masgrau R, Pujol A, Galea E. Staging anti-inflammatory therapy in Alzheimer’s disease. Front Aging Neurosci. 2010;2:142. 10.3389/fnagi.2010.00142. 21152343 10.3389/fnagi.2010.00142 PMC2998033

[43] Klunk WE, Engler H, Nordberg A, Wang Y, Blomqvist G, Holt DP, et al. Imaging brain amyloid in Alzheimer’s disease with Pittsburgh Compound-B. Ann Neurol. 2004;55(3):306–19. 10.1002/ana.20009. 14991808 10.1002/ana.20009

[44] Edison P, Archer HA, Hinz R, Hammers A, Pavese N, Tai YF, et al. Amyloid, hypometabolism, and cognition in Alzheimer disease: an [11C]PIB and [18F]FDG PET study. Neurology. 2007;68(7):501–8. 10.1212/01.wnl.0000244749.20056.d4. 17065593 10.1212/01.wnl.0000244749.20056.d4

[45] Kemppainen NM, Aalto S, Wilson IA, Nagren K, Helin S, Bruck A, et al. Voxel-based analysis of PET amyloid ligand [11C]PIB uptake in Alzheimer disease. Neurology. 2006;67(9):1575–80. 10.1212/01.wnl.0000240117.55680.0a. 16971697 10.1212/01.wnl.0000240117.55680.0a

[46] Okello A, Koivunen J, Edison P, Archer HA, Tukheimer FE, Nagren K, et al. Conversion of amyloid-positive and -negative MCI to AD over 3 years: an 11C-PIB PET study. Neurology. 2009;73(10):754–60. 10.1212/WNL.0b013e3181b23564. 19587325 10.1212/WNL.0b013e3181b23564 PMC2830881

[47] Braak H, Braak E. Neuropathological stageing of Alzheimer-related changes. Acta Neuropathol. 1991;82(4):239–59. 10.1007/BF00308809. 1759558 10.1007/BF00308809

[48] Sanchez JS, Becker JA, Jacobs HIL, Hanseeuw BJ, Jiang S, Schultz AP, et al. The cortical origin and initial spread of medial temporal tauopathy in Alzheimer’s disease assessed with positron emission tomography. Sci Transl Med. 2021;13(577):eabc0655. 10.1126/scitranslmed.abc0655. 33472953 10.1126/scitranslmed.abc0655 PMC7978042

[49] Pan N, Liu S, Ge X, Zheng Y, for Alzheimer’s Disease Neuroimaging Initiative. Association of hippocampal atrophy with tau pathology of temporal regions in preclinical Alzheimer’s disease. J Alzheimers Dis. 2025;104(1):13872877251314785. 10.1177/13872877251314785. 10.1177/13872877251314785 39956951

[50] Bellaver B, Povala G, Ferreira PCL, Ferrari-Souza JP, Leffa DT, Lussier FZ, et al. Astrocyte reactivity influences amyloid-β effects on tau pathology in preclinical Alzheimer’s disease. Nat Med. 2023;29:1775–81. 10.1038/s41591-023-02380-x. 37248300 10.1038/s41591-023-02380-x PMC10353939

[51] Lowe VJ, Lundt ES, Senjem ML, Schwarz CG, Min H-K, Przybelski SA, et al. White matter reference region in PET studies of 11 C-Pittsburgh Compound B uptake: effects of age and amyloid-β deposition. J Nucl Med. 2018;59(10):1583–9. 10.2967/jnumed.118.216114. 29674420 10.2967/jnumed.117.204271 PMC6167534

[52] Chiao P, Bedell BJ, Avants B, Zijdenbos AP, Grand’Maison M, O’Neill P, et al. Impact of reference and target region selection on amyloid PET SUV ratios in the Phase 1b PRIME study of aducanumab. J Nucl Med. 2019;60(1):100–6. 10.2967/jnumed.118.209130. 29777003 10.2967/jnumed.118.209130

[53] Petersen RC, Caracciolo B, Brayne C, Gauthier S, Jelic V, Fratiglioni L. Mild cognitive impairment: a concept in evolution. J Intern Med. 2014;275(3):214–28. 10.1111/joim.12190. 24605806 10.1111/joim.12190 PMC3967548

