# Supplementary material for: Astrocyte reactivity across the AD continuum measured by [18F]SMBT-1 and its relationship with the Aβ burden
Source: Eur J Nucl Med Mol Imaging. 2026 May 6;53(10):5659–74. doi: 10.1007/s00259-026-07824-3 (PMC13421302; doi:10.1007/s00259-026-07824-3)
Supplement: Supplementary file 1 — Supplementary Material 1 (DOCX 1.64 MB) [file 259_2026_7824_MOESM1_ESM.docx]

**Supplementary Information**

**Online Resource 1**

Inclusion and exclusion criteria of this study.

**Online Resource 2**

Comparisons of SUV between HC-, HC+, MCI-, MCI+ and AD (p-values)

**Online Resource 3**

Group comparison of raw SUV in candidate reference regions.

**Online Resource 4**

Group comparison of regional [^18^F]SMBT-1 SUVR using three alternative reference regions.

**Online Resource 5**

Comparison of regional [^18^F]SMBT-1 SUVR in the whole HC group versus amyloid-positive MCI and AD groups (reference region: CWM).

**Online Resource 6**

Voxel-wise SPM results of group comparisons based on [¹⁸F]SMBT-1 SUVR images

**Online Resource 7**

Voxel-wise group differences in [¹⁸F]SMBT-1 uptake for whole HC vs. MCI+ and whole HC vs. AD.

**Online Resource 1. Inclusion and exclusion criteria of this study.**

| **Group** | **Inclusion Criteria** | **Exclusion Criteria** |
| --- | --- | --- |
| MCI | (1) Informed consent by the participant or by the substitute (native Japanese) (2) Ages: 50 to 90 years old upon first examinations (3) Those who are diagnosed as MCI based on Petersen's criteria (4) Those who can tolerate relatively long PET examination (up to 45 min) | (1) Other neurological disorders (including cerebrovascular disorders, etc.), drug addicts, alcoholism, as well as severe cardiovascular diseases (2) Administration of irreversible MAO-B inhibitors (3) Habitual smoking (within 1 year of entry) (4) Being in the period of pregnancy, possible pregnancy, breast feeding, within 28 days after child birth (5) Severe allergic reactions to drugs and foods (including severe ethanol sensitivity: in Tohoku area only) (6) Psychiatric disorders, which may make the participation to this study difficult (7) Taboo conditions for MRI examinations (cardiac pacemaker, intracorporeal metals, etc. to be checked with questionnaire in advance) (8) Systemic circulatory and metabolic disorders as well as organ dysfunctions, such as diabetes mellites, hypertension, endocrinological disorders, congestive heart failure, angina pectoris, severe renal dysfunctions with Ccr<30mL/min or under plasmapheresis) (9) Past history of malignant diseases within 1 year of entry (*Early cancer patients with "minimal" effects due to therapeutic interventions, as confirmed by the responsible doctor, can be exceptionally included to the current study.) (10) Any sociomedical conditions, which may make the participation to this study difficult |
| AD | (1) Informed consent by the participant or by the substitute (native Japanese) (2) Ages: 50 to 90 years old upon first examinations (3) Those who are diagnosed as AD (4) Those who can tolerate relatively long PET examination (up to 45 min) | (1) Other neurological disorders (including cerebrovascular disorders, etc.), drug addicts, alcoholism, as well as severe cardiovascular diseases (2) Administration of irreversible MAO-B inhibitors (3) Habitual smoking (within 1 year of entry) (4) Being in the period of pregnancy, possible pregnancy, breast feeding, within 28 days after child birth (5) Severe allergic reactions to drugs and foods (including severe ethanol sensitivity: in Tohoku area only) (6) Psychiatric disorders, which may make the participation to this study difficult (7) Taboo conditions for MRI examinations (cardiac pacemaker, intracorporeal metals, etc. to be checked with questionnaire in advance) (8) Systemic circulatory and metabolic disorders as well as organ dysfunctions, such as diabetes mellites, hypertension, endocrinological disorders, congestive heart failure, angina pectoris, severe renal dysfunctions with Ccr<30mL/min or under plasmapheresis) (9) Past history of malignant diseases within 1 year of entry (*Early cancer patients with "minimal" effects due to therapeutic interventions, as confirmed by the responsible doctor, can be exceptionally included to the current study.) (10) Any sociomedical conditions, which may make the participation to this study difficult |
| HC | (1) Informed consent by the participant or by the substitute (native Japanese) (2) Ages: basically 50 to 90 years old upon first examinations (3) MMSE scores higher than or equal to 24 (4) Wechsler memory Scale-R Logical memory II scores higher than cut-off (>3 for those who have education period of 0-7 years; >5 for education period of 8-15 years; >9 for education period of more than 16 years) (5) Those who can tolerate relatively long PET examination (up to 2 hours) | (1) Any neurological disorders (2) drug addicts including alcoholism (3) Habitual smoking (within 1 year of entry) (4) Being in the period of pregnancy, possible pregnancy, breast feeding, within 28 days after child birth (5) Severe allergic reactions to drugs and foods (including severe ethanol sensitivity: in Tohoku area only) (6) Psychiatric disorders, which may make the participation to this study difficult (7) Taboo conditions for MRI examinations (cardiac pacemaker, intracorporeal metals, etc. to be checked with questionnaire in advance) (8) Systemic circulatory and metabolic disorders as well as organ dysfunctions, such as diabetes mellites, hypertension, endocrinological disorders, congestive heart failure, angina pectoris, severe renal dysfunctions with Ccr<30mL/min or under plasmapheresis) (9) Past history of malignant diseases within 1 year of entry (*Early cancer patients with "minimal" effects due to therapeutic interventions, as confirmed by the responsible doctor, can be exceptionally included to the current study.) (10) Any sociomedical conditions, which may make the participation to this study difficult |

**Online Resource 2. Comparisons of SUV between HC-, HC+, MCI-, MCI+ and AD (p-values)**

| Reference | HC- | HC+ | MCI- | MCI+ | AD | method | overall p value |
| --- | --- | --- | --- | --- | --- | --- | --- |
| CGM | 1.24 ± 0.25 | 1.22 ± 0.17 | 1.27 ± 0.25 | 1.19 ± 0.31 | 1.13 ± 0.27 | ANOVA | 0.605 |
| CWM | 1.67 ± 0.29 | 1.71 ± 0.25 | 1.71 ± 0.27 | 1.60 ± 0.38 | 1.48 ± 0.27 | ANOVA | 0.282 |
| SWM | 1.86 ± 0.30 | 1.94 ± 0.42 | 1.97 ± 0.30 | 1.91 ± 0.43 | 1.82 ± 0.32 | ANOVA | 0.781 |
| WC | 1.34 ± 0.26 | 1.34 ± 0.18 | 1.37 ± 0.25 | 1.28 ± 0.32 | 1.22 ± 0.25 | ANOVA | 0.524 |

Values are presented as mean ± standard deviation (SD) of the raw Standardized Uptake Value (SUV). The “overall p” column displays the p-value from a one-way Analysis of Variance (ANOVA) comparing the mean SUV across the five diagnostic groups for each reference region. A p-value > 0.05 indicates no significant difference between the groups, supporting the stability of the reference region.

Abbreviations: CGM, cerebellar gray matter; CWM, cerebellar white matter; SWM, subcortical white matter; WC, whole cerebellum; HC-, amyloid-negative healthy controls; HC+, amyloid-positive healthy controls; MCI-, amyloid-negative mild cognitive impairment; MCI+, amyloid-positive mild cognitive impairment; AD, Alzheimer's disease.

**Online Resource 3. Group comparison of raw SUV in candidate reference regions.**

| Reference | contrast | estimate | SE | df | t.ratio | p.value |
| --- | --- | --- | --- | --- | --- | --- |
| CGM | HC-vs HC+ | 0.03 | 0.11 | 86 | 0.25 | > 0.99 |
|  | HC- vs MCI- | -0.03 | 0.08 | 86 | -0.38 | > 0.99 |
|  | HC-vs MCI+ | 0.06 | 0.07 | 86 | 0.77 | > 0.99 |
|  | HC- vs AD | 0.11 | 0.09 | 86 | 1.23 | > 0.99 |
|  | HC+ vs MCI- | -0.06 | 0.11 | 86 | -0.51 | > 0.99 |
|  | HC+ vs MCI+ | 0.03 | 0.11 | 86 | 0.28 | > 0.99 |
|  | HC+ vs AD | 0.09 | 0.12 | 86 | 0.71 | > 0.99 |
|  | MCI- vs MCI+ | 0.09 | 0.08 | 86 | 1.07 | > 0.99 |
|  | MCI- vs AD | 0.14 | 0.10 | 86 | 1.47 | > 0.99 |
|  | MCI+ vs AD | 0.06 | 0.09 | 86 | 0.61 | > 0.99 |
| CWM | HC- vs HC+ | -0.04 | 0.12 | 86 | -0.30 | > 0.99 |
|  | HC- vs MCI- | -0.04 | 0.09 | 86 | -0.45 | > 0.99 |
|  | HC- vs MCI+ | 0.07 | 0.09 | 86 | 0.79 | > 0.99 |
|  | HC- vs AD | 0.19 | 0.11 | 86 | 1.75 | 0.83 |
|  | HC+ vs MCI- | 0.00 | 0.13 | 86 | -0.03 | > 0.99 |
|  | HC+ vs MCI+ | 0.11 | 0.13 | 86 | 0.84 | > 0.99 |
|  | HC+ vs AD | 0.23 | 0.14 | 86 | 1.60 | > 0.99 |
|  | MCI- vs MCI+ | 0.11 | 0.09 | 86 | 1.16 | > 0.99 |
|  | MCI- vs AD | 0.23 | 0.11 | 86 | 2.01 | 0.48 |
|  | MCI+ vs AD | 0.12 | 0.11 | 86 | 1.10 | > 0.99 |
| SWM | HC- vs HC+ | -0.09 | 0.14 | 86 | -0.61 | > 0.99 |
|  | HC- vs MCI- | -0.11 | 0.11 | 86 | -1.04 | > 0.99 |
|  | HC- vs MCI+ | -0.05 | 0.10 | 86 | -0.52 | > 0.99 |
|  | HC-vs AD | 0.03 | 0.12 | 86 | 0.25 | > 0.99 |
|  | HC+ vs MCI- | -0.02 | 0.15 | 86 | -0.16 | > 0.99 |
|  | HC+ vs MCI+ | 0.04 | 0.14 | 86 | 0.25 | > 0.99 |
|  | HC+ vs AD | 0.12 | 0.16 | 86 | 0.73 | > 0.99 |
|  | MCI- vs MCI+ | 0.06 | 0.11 | 86 | 0.55 | > 0.99 |
|  | MCI-vs AD | 0.14 | 0.13 | 86 | 1.09 | > 0.99 |
|  | MCI+ vs AD | 0.08 | 0.12 | 86 | 0.66 | > 0.99 |
| WC | HC- vs HC+ | 0.01 | 0.11 | 86 | 0.06 | > 0.99 |
|  | HC- vs MCI- | -0.03 | 0.08 | 86 | -0.36 | > 0.99 |
|  | HC- vs MCI+ | 0.07 | 0.08 | 86 | 0.90 | > 0.99 |
|  | HC- vs AD | 0.12 | 0.09 | 86 | 1.32 | > 0.99 |
|  | HC+ vs MCI- | -0.04 | 0.11 | 86 | -0.31 | > 0.99 |
|  | HC+ vs MCI+ | 0.06 | 0.11 | 86 | 0.56 | > 0.99 |
|  | HC+ vs AD | 0.12 | 0.12 | 86 | 0.95 | > 0.99 |
|  | MCI- vs MCI+ | 0.10 | 0.08 | 86 | 1.17 | > 0.99 |
|  | MCI- vs AD | 0.15 | 0.10 | 86 | 1.53 | > 0.99 |
|  | MCI+ vs AD | 0.06 | 0.10 | 86 | 0.59 | > 0.99 |

Statistical analysis was performed using one-way Analysis of Variance (ANOVA) for each reference region to test for an overall group effect, followed by Bonferroni correction test for post-hoc pairwise comparisons. Degrees of freedom (df) = 86 in all comparisons. Values for the estimate (mean difference), standard error (SE),t-ratio, and p-value are rounded to two decimal places. P values greater than 0.99 are reported as ‘> 0.99’.

Abbreviations: AD, Alzheimer's disease; CGM, cerebellar gray matter; CWM, cerebellar white matter; HC, amyloid-negative healthy controls; HC+, amyloid-positive healthy controls; MCI-, amyloid-negative mild cognitive impairment; MCI+, amyloid-positive mild cognitive impairment; SWM, subcortical white matter; WC, whole cerebellum.

**Online Resource 4. Group comparison of regional [^18^F]SMBT-1 SUVR using three alternative reference regions.**


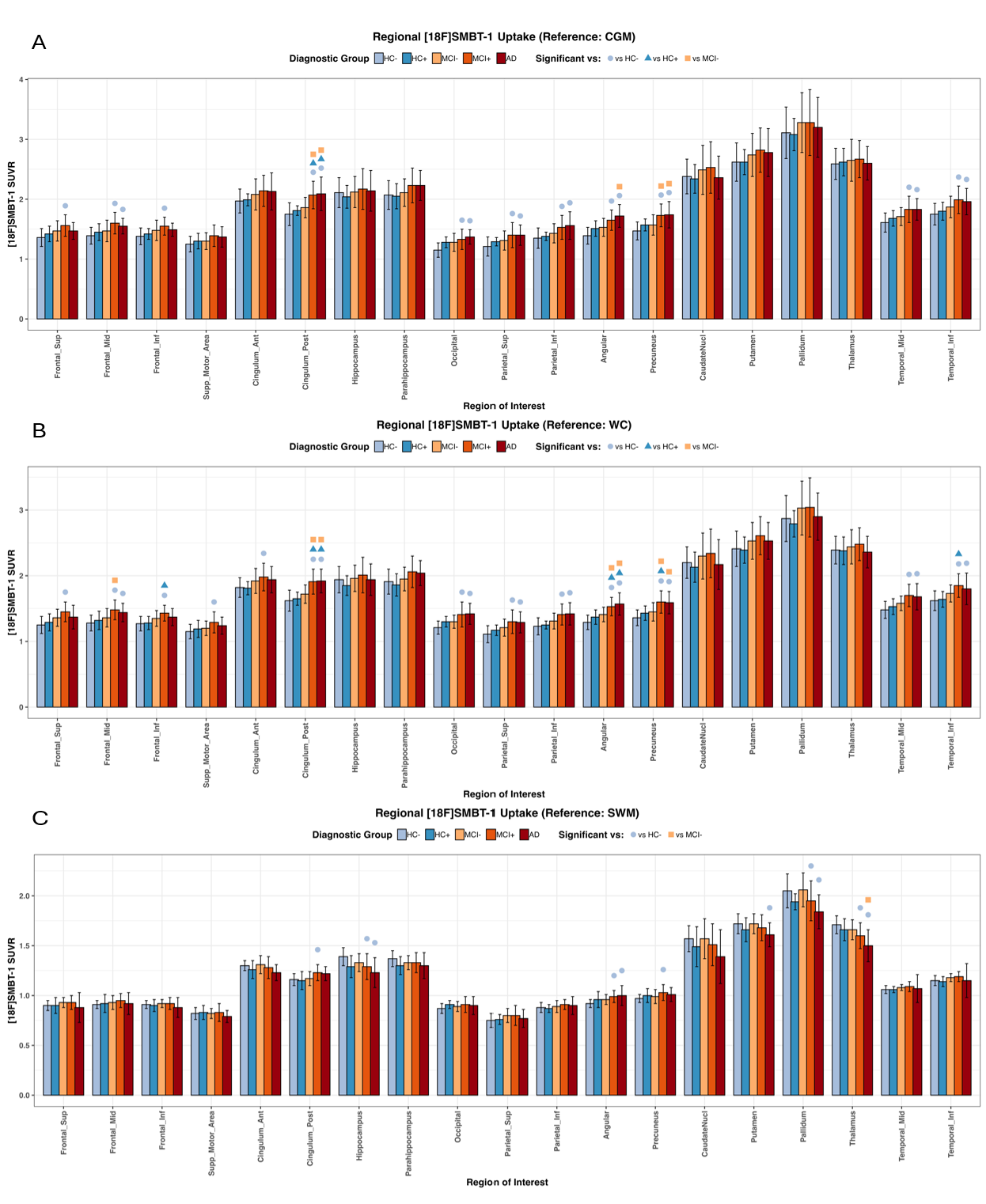


Mean [¹⁸F]SMBT-1 Standardized Uptake Value Ratio (SUVR) values were calculated and presented for the five diagnostic groups. Error bars denote the standard deviation. The results are based on three different reference regions: (A) cerebellar gray matter, (B) whole cerebellum, and (C) subcortical cerebellar white matter. Representative 19 regions with highest statistical significances were selected from the whole-brain ROI analysis for clear visualization of the main findings, including 49 regions with statistical significance out of 95 regions.

Significance symbols: A blue circle (●) indicates a significant difference versus the HC− group; a green triangle (▲) indicates a significant difference versus the HC+ group; and an orange square (■) indicates a significant difference versus the MCI− group.

Abbreviations: AD, Alzheimer’s disease; CGM, cerebellar gray matter; HC Aβ-, amyloid-β negative healthy controls; HC Aβ+, amyloid-β positive healthy controls; MCI Aβ-, amyloid-β negative mild cognitive impairment; MCI Aβ+, amyloid-β positive mild cognitive impairment; SWM, subcortical white matter; SUVR, Standardized Uptake Value Ratio; WC, whole cerebellum.

Abbreviations: AD, Alzheimer’s disease; CGM, cerebellar gray matter; CWM, cerebellar white matter; WC, whole cerebellum; Amygdala, Amygdala; SWM, subcortical white matter; Angular, Angular Gyrus; Calcarine, Calcarine Cortex; CaudateNucl, Caudate Nucleus; Cingulum_Ant, Anterior Cingulate Gyrus; Cingulum_Post, Posterior Cingulate Gyrus; Frontal_Inf, Inferior Frontal Gyrus; Frontal_Mid, Middle Frontal Gyrus; Frontal_Sup, Superior Frontal Gyrus; Insula, Insular Cortex; Lingual, Lingual Gyrus; Parietal_Inf, Inferior Parietal Lobule; Parietal_Sup, Superior Parietal Lobule; Supp_Motor_Area, Supplementary Motor Area; Supra_Marginal, Supramarginal Gyrus; Temporal_Inf, Inferior Temporal Gyrus; Temporal_Mid, Middle Temporal Gyrus; Temporal_Sup, Superior Temporal Gyrus.

**Online Resource 5.** **Comparison of regional [^18^F]SMBT-1 SUVR in the whole HC group versus amyloid-positive MCI and AD groups (reference region: CWM).**

|  | **HC** | **MCI Ab +** | **p value** | **AD** | **p value** |
| --- | --- | --- | --- | --- | --- |
| Frontal_Sup | 1.26 ± 0.12 | 1.41 ± 0.13 | p<0.001 | 1.41 ± 0.15 | p=0.002 |
| Frontal_Mid | 1.02 ± 0.09 | 1.17 ± 0.11 | p<0.001 | 1.18 ± 0.14 | p<0.001 |
| Frontal_Inf | 1.12 ± 0.09 | 1.25 ± 0.12 | p<0.001 | 1.25 ± 0.11 | p=0.004 |
| Supp_Motor_Area | 0.92 ± 0.09 | 1.02 ± 0.13 | p=0.013 | 1.01 ± 0.12 | p=0.022 |
| Cingulum_Ant | 1.44 ± 0.10 | 1.57 ± 0.13 | p=0.003 | 1.59 ± 0.17 | p=0.001 |
| Cingulum_Post | 1.26 ± 0.10 | 1.47 ± 0.14 | p<0.001 | 1.48 ± 0.12 | p<0.001 |
| Hippocampus | 1.53 ± 0.13 | 1.59 ± 0.17 |  | 1.58 ± 0.16 |  |
| Parahippocampus | 1.51 ± 0.12 | 1.63 ± 0.16 | p=0.008 | 1.67 ± 0.15 | p=0.001 |
| Occipital | 0.98 ± 0.09 | 1.11 ± 0.13 | p<0.001 | 1.16 ± 0.14 | p<0.001 |
| Parietal_Sup | 0.90 ± 0.10 | 1.03 ± 0.14 | p=0.001 | 1.06 ± 0.13 | p=0.001 |
| Parietal_Inf | 0.98 ± 0.09 | 1.12 ± 0.12 | p<0.001 | 1.16 ± 0.13 | p=0.002 |
| Angular | 1.04 ± 0.09 | 1.21 ± 0.12 | p<0.001 | 1.28 ± 0.15 | p<0.001 |
| Precuneus | 1.07 ± 0.09 | 1.24 ± 0.14 | p<0.001 | 1.26 ± 0.11 | p<0.001 |
| CaudateNucl | 1.74 ± 0.17 | 1.85 ± 0.24 |  | 1.77 ± 0.33 |  |
| Putamen | 1.91 ± 0.18 | 2.06 ± 0.17 | p=0.043 | 2.07 ± 0.26 | p=0.009 |
| Pallidum | 2.27 ± 0.23 | 2.40 ± 0.28 |  | 2.37 ± 0.31 |  |
| Thalamus | 1.90 ± 0.14 | 1.96 ± 0.13 |  | 1.93 ± 0.24 |  |
| Temporal_Mid | 1.19 ± 0.10 | 1.34 ± 0.11 | p<0.001 | 1.38 ± 0.18 | p<0.001 |
| Temporal_Inf | 1.29 ± 0.10 | 1.46 ± 0.11 | p<0.001 | 1.48 ± 0.21 | p<0.001 |

This table presents the mean standardized uptake value ratio (SUVR) standard deviation (SD) for the combined Entire Healthy Control (HC) cohort (HC- and HC+), MCI+ group, and AD group across selected Regions of Interest (ROIs). SUVR was calculated using the cerebellar white matter (CWM) as the reference region. Statistical comparisons were performed using ANOVA followed by post-hoc analysis to compare the HC group against the MCI+ and AD groups. P-values represent the statistical significance of the difference between the HC group and the respective amyloid-positive group (MCI+) or (AD). Significant differences (P < 0.05) are highlighted.

Abbreviations: AD, Alzheimer’s disease; CGM, cerebellar gray matter; CWM, cerebellar white matter; WC, whole cerebellum; Amygdala, Amygdala; SWM, subcortical white matter; Angular, Angular Gyrus; Calcarine, Calcarine Cortex; CaudateNucl, Caudate Nucleus; Cingulum_Ant, Anterior Cingulate Gyrus; Cingulum_Post, Posterior Cingulate Gyrus; Frontal_Inf, Inferior Frontal Gyrus; Frontal_Mid, Middle Frontal Gyrus; Frontal_Sup, Superior Frontal Gyrus; Insula, Insular Cortex; Lingual, Lingual Gyrus; Parietal_Inf, Inferior Parietal Lobule; Parietal_Sup, Superior Parietal Lobule; Supp_Motor_Area, Supplementary Motor Area; Supra_Marginal, Supramarginal Gyrus; Temporal_Inf, Inferior Temporal Gyrus; Temporal_Mid, Middle Temporal Gyrus; Temporal_Sup, Superior Temporal Gyrus.

**Online Resource 6.** **Voxel-wise SPM results of group comparisons based on [¹⁸F]SMBT-1 SUVR images**

| Comparison | Brain Regions | Peak in MNI Coordinates (x,y,z) | Cluster Size  (kE​) | Peak T-score | Peak Z-score | p value (uncorr) |  |
| --- | --- | --- | --- | --- | --- | --- | --- |
| HC- < MCI+ | Precuneus / PCC | (-2, -66, 28) | 4936 | 4.52 | 4.27 | < 0.001 |  |
|  | Angular Gyrus | (44, 46, -4) | 2728 | 4.31 | 4.09 | < 0.001 |  |
|  | Superior Frontal Gyrus | (-38, 50, -6) | 353 | 3.92 | 3.75 | < 0.001 |  |
|  | Middle Temporal Gyrus | (60, -32, -26) | 549 | 3.86 | 3.70 | < 0.001 |  |
| HC- < AD | Precuneus / PCC | (26, -68, 44) | 2709 | 3.68 | 3.54 | < 0.001 |  |
|  | Middle Temporal Gyrus | (-40, -62, 30) | 3023 | 3.59 | 3.46 | < 0.001 |  |
|  | Angular Gyrus | (44, -54, 36) | [*part of MTG cluster] | 3.53 | 3.40 | < 0.001 |  |
| MCI- < MCI+ | Precuneus/ PCC | (-2, -70, 32) | [*part of MTG cluster] | 3.44 | 3.32 | < 0.001 |  |
|  | Superior Frontal Gyrus | (0, 60, -66) | 2555 | 5.02 | 4.69 | < 0.001 |  |
|  | Occipital / Cuneus | (50, 12, 34) | 388 | 4.25 | 4.04 | < 0.001 |  |
|  |  |  |  |  |  |  |  |
| HC_whole_ < MCI+ | Precuneus / PCC | (-2, -64, 28) | 8508 | 4.58 | 4.32 | < 0.001 |  |
|  | Angular Gyrus | (44, 46, -6) | 4128 | 4.51 | 4.26 | < 0.001 |  |
|  | Middle Temporal Gyrus | (60, -32, -28) | 1070 | 4.33 | 4.11 | < 0.001 |  |
|  | Superior Frontal Gyrus | (-38, 50, -8) | 2216 | 4.17 | 3.97 | < 0.001 |  |
| HC_whole_ < AD | Precuneus / PCC | (26, -68, 44) | 2608 | 3.67 | 3.53 | < 0.001 |  |
|  | Middle Temporal Gyrus | (-40, -62, 30) | 2706 | 3.63 | 3.49 | < 0.001 |  |
|  | Inferior Temporal Gyrus | (-54, -28, -18) | 674 | 3.59 | 3.45 | < 0.001 |  |
|  | Angular Gyrus | (42, -54, 34) | [*part of MTG cluster] | 3.53 | 3.41 | < 0.001 |  |
|  | Frontal Mid Gyrus | (-28, 8, 42) | 430 | 3.40 | 3.28 | 0.001 |  |

Voxel-wise statistical parametric mapping (SPM) analyses were performed using SUVR images normalized to the cerebellar white matter (CWM). Reported regions correspond to peak coordinates within significant clusters (voxel-level threshold p < 0.001, uncorrected). Cluster size (kE) is reported at the cluster level. Regions labeled as “*part of cluster” represent local maxima within a larger contiguous cluster.

Abbreviations: HC-, amyloid-negative healthy controls; AD, Alzheimer’s disease; MCI+, amyloid-positive mild cognitive impairment; MCI-, amyloid-negative mild cognitive impairment; HC_whole_, whole healthy controls (amyloid-negative and amyloid-positive combined); MTG, middle temporal gyrus.

**Online Resource 7. Voxel-wise group differences in [¹⁸F]SMBT-1 uptake for whole HC vs. MCI+ and whole HC vs. AD.**


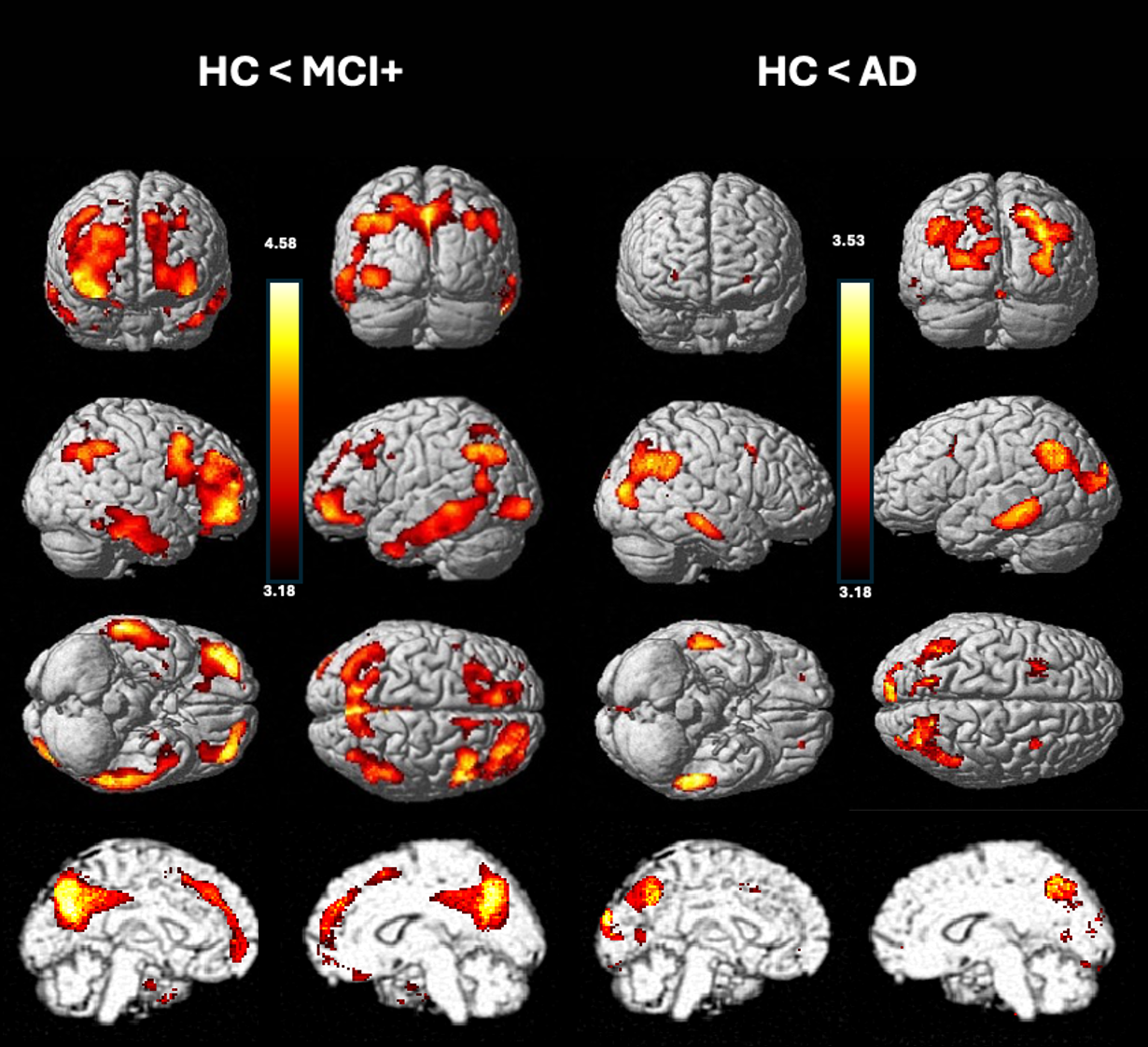


Statistical parametric maps displayed on a standard brain template, illustrating regions of significantly higher [¹⁸F]SMBT-1 uptake in MCI+ compared with the entire healthy control group (HC) (Left: “HC < MCI+”), as well as those in AD compared with HC (Right “HC < AD”).

The HC < MCI contrast is greater than the HC < AD contrast partly because of a non-linear astrocyte response in different disease stages from MCI to AD, partial-volume effects, and differences in sample sizes between the MCI (n = 25) and AD (n = 12) subgroups. The color bar represents T-values. Three-dimensional (3D) rendered images were generated by Statistical parametric mapping (SPM) software package, in which 2 types of 3D rendered images are combined in this figure. Surface-rendered images are default style of SPM12 (TOP three rows), and medial brain image is shown in SPM96-like style (BOTTOM), both generated by SPM12. Statistical threshold: p < 0.001, uncorrected, no cluster extent threshold.

Abbreviations: AD, Alzheimer’s disease; HC, healthy controls (amyloid-negative and amyloid-positive combined); MCI+, amyloid-positive mild cognitive impairment.
